# The threat of multidrug-resistant microorganisms: active surveillance of key antimicrobial resistant pathogens in 2025 - a report from the INVIFAR network

**DOI:** 10.1007/s10096-025-05330-2

**Published:** 2026-01-06

**Authors:** Adrián Martínez-Meléndez, Elvira Garza-González, María del Rosario Vázquez-Larios, Melissa Garibaldi-Rojas, Bernardo Alfonso Martinez-Guerra, Christian Daniel Mireles-Davalos, Samuel Pavel Escalante-Armenta, José Manuel Feliciano-Guzmán, Daniel Romero-Romero, Maria del Consuelo Velazquez-Acosta, Sandra Quintana-Ponce, Shaúl Ariel Navarro-Lara, Jesús Alfonso Aguirre-Torres, María Guadalupe Martínez-Zavaleta, Ana Karina Castillo-Perez, Juan Pablo Mena-Ramírez, Elena Victoria Choy-Chang, Laura Karina Avilés-Benítez, María Guadalupe Fong-Camargo, Carlos Antonio Couoh-May, Eduardo López-Gutiérrez, Talia Pérez-Vicelis, Aldo Rafael Silva-Gamiño, Joaquín Rincón-Zuno, Mariana Gil-Veloz, Héctor Miguel Zubiate-Tejeda, Eloisa Ramirez-Alanis, Maricruz Gutierrez-Brito, Josue Gomez-Espinosa, Ricardo García-Romo, Juan Manuel Barajas-Magallón, Cecilia Teresita Morales-de-la-Peña, Guillermo Jacobo-Baca, María Bertha Ballesteros-Silva, Paola Alejandra Preciado-Jiménez, Luis David Chora-Hernández, Isabel Cristina Márquez-Avalos, Hiram Villanueva-Lozano, Enrique Bolado-Martínez, Juan de Dios Castañeda-Duarte, Cecilia Padilla-Ibarra, Victor Hugo Peralta-Peñuñuri, Lizbeth Soraya Duarte-Miranda, Anabel Valenzuela-Oroz, Angela Cecilia Valtierra-Diosdado, Paulina Fabiola González-Melgoza, Jorge Arturo Salazar-Mares, Diana Eugenia Perales-Martínez, Marliz Andrea Vazquez-Diaz, Guadalupe Soledad Huirache-Villalobos, Filiberto Alejandro Martínez-Lazo, Margarita Alcaraz-Espejel, Rodrigo E. Vázquez-Olvera, Martha Dorado-del-Rio, Iván Ramón Pérez-Méndez, Zaira Lucero Clemente-Callejas, Juana Narmy Cardona-Olguin, Elisa Sánchez-García, Paola Bocanegra-Ibarias, Rafael Franco-Cendejas, Luis Esaú López-Jácome

**Affiliations:** 1 Facultad de Ciencias Químicas, Universidad Autónoma de Nuevo León, Monterrey, Nuevo León 66455 Mexico; 2 Departamento de Bioquímica y Medicina Molecular, Facultad de Medicina, Universidad Autónoma de Nuevo León, Monterrey, Nuevo León 66460 Mexico; 3 Instituto Nacional de Cardiología “Ignacio Chávez”, Ciudad de México, 14080 Mexico; 4 Instituto Nacional de Ciencias Médicas y Nutrición “Salvador Zubirán”, Ciudad de México, 14080 Mexico; 5 Instituto Nacional de Enfermedades Respiratorias “Ismael Cosío Villegas”, Ciudad de México, 14080 Mexico; 6 Hospital General y Hospital del niño y la mujer (IMSS Bienestar), Ciudad Obregón, 85000 Sonora, Mexico; 7 Especialidades Pediátricas IMSS-Bienestar, Tuxtla Gutiérrez, Chiapas 29070 Mexico; 8 Diagnóstico Médico Integral “Pasteur”, Toluca, Estado de México 50020 Mexico; 9 Instituto Nacional de Cancerología, Ciudad de México, 14480 Mexico; 10 Facultad de Ciencias Naturales, Universidad Autónoma de Guerrero, Chilpancingo, Guerrero 39070 Mexico; 11 Laboratorio de Microbiología, Hospital Civil “Fray Antonio Alcalde”, Guadalajara, Jalisco 44280 Mexico; 12 Hospital del Niño Morelense, Emiliano Zapata, Morelos 62765 Mexico; 13 Laboratorio de Microbiología Clínica, Instituto Nacional de Rehabilitación Luis Guillermo Ibarra Ibarra, Ciudad de México, 14389 Mexico; 14 Hospital “Dr, Manuel GEA González”, Ciudad de México, 14080 Mexico; 15 Hospital General de Zona No. 21, 47630 Tepatitlán de Morelos, Jalisco Mexico; 16 Hospital General de Zona No, IMSS Nueva Frontera, Tapachula, Chiapas 30767 Mexico; 17 Hospital Infantil de Morelia, Morelia, Michoacán 58253 Mexico; 18 Hospital General de Zona No. 35, Juárez, Chihuahua 32809 Mexico; 19 Hospital General “Dr, Agustín O´horan”, Mérida, Yucatán 97300 Mexico; 20 Hospital Regional de Alta Especialidad de Oaxaca, San Bartolo Coyotepec, Oaxaca 71294 Mexico; 21 Hospital Regional de Alta Especialidad “Bicentenario de la Independencia”, Tultitlán, Estado de México 54916 Mexico; 22 Hospital Ángeles Morelia, Morelia, Michoacán 58350 Mexico; 23 Hospital para el Niño de Toluca IMIEM, Toluca, Estado de México 50170 Mexico; 24 Hospital Regional de Alta Especialidad del Bajío, 37660 León, Guanajuato Mexico; 25 Hospital General “Lázaro Cárdenas”, 31203 Chihuahua, Chihuahua Mexico; 26 Departamento de, Microbiología del Hospital la Luz, Morelia, Michoacán 58260 Mexico; 27 Hospital de la Niñez Poblana, San Andrés Cholula, Puebla 72190 Mexico; 28 Hospital Chiapas Nos Une. “Dr. Jesus Gilberto Gomez Maza”, Tuxtla Gutiérrez, Chiapas 29045 Mexico; 29 Centenario Hospital Miguel Hidalgo, Aguascalientes, Aguascalientes 20259 Mexico; 30 Laboratorio DIPROMI, Morelia, Michoacán 58260 Mexico; 31 Hospital General “Juan María de Salvatierra”, La Paz, Baja California Sur 23080 Mexico; 32 Centro Universitario de Salud, Universidad Autónoma de Nuevo León, Monterrey, Nuevo León 67110 Mexico; 33 Centro de diagnóstico microbiológico SA de CV, Morelia, Michoacán 58000 Mexico; 34 Laboratorios del Centro, Zamora, Michoacán 59600 Mexico; 35 Hospital General “Dr. Miguel Silva”, Morelia, Michoacán 58253 Mexico; 36 Hospital del niño “Dr, Federico Gómez Santos”, Saltillo, Coahuila 25022 Mexico; 37 Hospital Regional “Alta Especialidad” ISSSTE, Monterrey, Nuevo León 64380 Mexico; 38 Universidad de Sonora, Hermosillo, Sonora 83000 Mexico; 39 Centro Médico “Dr, Ignacio Chávez”, Hermosillo, Sonora 83000 Mexico; 40 Hospital General de Estado de Sonora, Hermosillo, Sonora 83000 Mexico; 41 HG “Dr. Fernando Ocaranza”, Hermosillo, Sonora 83150 Mexico; 42 Centro Integral de Atención a la Salud Unidad Sur ISSSTESON, Hermosillo, Sonora 83200 Mexico; 43 Hospital “Adolfo López Mateos”-ISSSTESON, 85000 Ciudad Obregón, Sonora Mexico; 44 Hospital General León, 37670 León, Guanajuato Mexico; 45 Laboratorio Futura Medica, Morelia, Michoacán 58280 Mexico; 46 Hospital General Silao, Silao, Guanajuato 36133 Mexico; 47 Hospital General ISSSTE San Luis Potosí, San Luis Potosí, San Luis Potosí, 78339 Mexico; 48 HGZ #46 IMSS Tabasco, Villahermosa, Tabasco 86160 Mexico; 49 Laboratorio Clínico y Bacteriológico LB, La Barca, Jalisco, 47913 Mexico; 50 Bio Diagnostics Laboratorio Clínico SC, 90300 Apizaco, Tlaxcala Mexico; 51 Laboratorio SEMLAB, Morelia, Michoacán 58260 Mexico; 52 Hospital Regional “General Ignacio Zaragoza” ISSSTE, Ciudad de México, 09220 Mexico; 53 Laboratorio Dorado, Mexicali, Baja California 21100 Mexico; 54 Hospital Español Veracruz, Veracruz, Veracruz 91700 Mexico; 55 Hospital General de Querétaro, Querétaro, Querétaro 76180 Mexico; 56 Hospital General Regional No.6 “Dr. Ignacio García Téllez”, Ciudad Madero, Tamaulipas 89400 Mexico; 57 Lapi Laboratorio Médico, Ciudad de México, 01470 Mexico; 58 Hospital Universitario Dr, José Eleuterio González, Universidad Autónoma de Nuevo León, Nuevo León, 64460 Monterrey, Mexico; 59 Subdirección de Investigación Biomédica, Instituto Nacional de Rehabilitación Luis Guillermo Ibarra Ibarra, Ciudad de México, 14389 Mexico; 60 Departamento de Biología, Facultad de Química, Universidad Nacional Autónoma de México, Ciudad de México, 04510 Mexico

**Keywords:** Antimicrobial resistance, Enterobacterales, Pseudomonas aeruginosa, Acinetobacter baumannii, Clinical specimens, Bla_OXA−48−like_, *bla*_OXA24_, *bla*_NDM_

## Abstract

**Purpose:**

Systematic collection and analysis of antimicrobial resistance data from key bacterial pathogens is essential to contribute to control antimicrobial resistance (AMR). The aim of this work was to survey the drug resistance on clinically relevant organisms stratified according to age, gender, clinical specimens and facilities.

**Methods:**

Microbiological data were collected from 55 centers across 24 states in Mexico between January 1 and March 31, 2025. Bacterial identification and antimicrobial susceptibility testing were performed at each participating center using locally available methods. Data was processed using WHONET 2025. Isolates obtained from lower respiratory specimens, urine, blood, biopsies and abscesses were analyzed. Carbapenem non-susceptible isolates were further analyzed by PCR for common carbapenemase-encoding genes. Resistance frequencies were compared using the chi-square test.

**Results:**

A total of 11,290 clinical isolates were analyzed, mostly from urine (*n* = 7,149; 63.3%), followed by blood (*n* = 1,370; 12.1%). The most prevalent was *Escherichia coli* (*n* = 6,185; 54.8%), followed by *Klebsiella pneumoniae* (*n* = 1,365; 12.1%) and *Pseudomonas aeruginosa* (*n* = 1,110; 9.8%). Resistance to carbapenems in *E. coli* was higher in respiratory isolates (imipenem: 5.8%, *p* = 0.016; meropenem: 5.3%, *p* < 0.001), with 75.9% producing extended-spectrum ß-lactamases (ESBLs). *K. pneumoniae* had the highest resistance to ampicillin/sulbactam (52.5%, *p* = 0.028) and sulfamethoxazole/trimethoprim (62.1%, *p* = 0.014) in blood isolates, and 63.2% were ESBL-producers (*p* = 0.001). In *P. aeruginosa*, urine isolates showed significantly higher resistance to ceftolozane-tazobactam (24.7%, *p* = 0.008), ceftazidime-avibactam (36.6%, *p* < 0.001), and meropenem (34.5%, *p* = 0.009) compared to other clinical specimens included. For *A. baumannii*, respiratory isolates had 73.6% resistance to meropenem (*p* < 0.001). *S. aureus* from blood showed 25.7% resistance to oxacillin (*p* < 0.004). The most frequent carbapenemase genes were *bla*_OXA−48−like_ in *E. coli* (26/56, 46.4%), *bla*_NDM_ for *K. pneumoniae* (7/17, 41.2%), *bla*_OXA−24_ in *A. baumannii* (79/108, 73.1%) and *bla*_IMP_ for *P. aeruginosa* (18/108, 16.7%).

**Conclusion:**

This surveillance study underscores the elevated levels of antimicrobial resistance, ESBL production, and carbapenemase activity among priority pathogens, including some *Enterobacterales*, *P. aeruginosa*, and *A. baumannii*. These findings emphasize the urgent need to strengthen epidemiologic surveillance programs in Mexican healthcare settings.

**Supplementary Information:**

The online version contains supplementary material available at 10.1007/s10096-025-05330-2.

## Introduction

The global rise of antimicrobial resistance (AMR) represents one of the most urgent public health challenges of the 21 st century. Multidrug-resistant (MDR) microorganisms are increasingly responsible for healthcare-associated infections, contributing to elevated morbidity and mortality, prolonged hospital stays, and escalating healthcare costs [1].

Despite increasing awareness, the spread of resistant pathogens continues to outpace the development of new therapeutic options, especially in low- and middle-income countries where surveillance infrastructure remains limited [2, 3]. To counter this threat, coordinated and sustained surveillance systems are essential for detecting resistance trends, guiding public health interventions, and informing clinical decision-making [1].

The INVIFAR Network (Red Temática de Investigación y Vigilancia de la Farmacorresistencia) was established in Mexico in 2018 to address this need. It is a national collaborative platform that unites clinical laboratory scientists, microbiologists, epidemiologists, infectious disease specialists, clinical and research institutions to strengthen the surveillance and investigation of AMR. Since its inception, INVIFAR has enabled the systematic collection, analysis, and dissemination of resistance data from key bacterial pathogens of clinical importance. The network has conducted several multicenter studies, providing critical insights into resistance mechanisms, molecular epidemiology, and the emergence of high-risk clones in the Mexican healthcare setting, including some Gram negatives and *Staphylococcus aureus* [4–6]. These efforts have also contributed to evidence-based recommendations for hospital infection control and antimicrobial stewardship.

The systematic reporting of findings derived from active surveillance of AMR in high-priority pathogens is essential to guide the development of strategies to control AMR at INVIFAR-affiliated centers [7], The aim of this work was to survey the drug resistance on clinically relevant organisms stratified according to age, gender, clinical specimens and facilities in Mexico during a 3-month period.

## Methods

### Participating centers and data collection

Participating centers are members of the Network for the Research and Surveillance of Drug Resistance (Red Temática de Investigación y Vigilancia de la Farmacorresistencia, INVIFAR). Microbiological data—including species identification and antimicrobial susceptibility testing—were collected from clinical isolates obtained willingly, between January 1, 2025, and March 31, 2025.

Bacterial identification and antimicrobial susceptibility testing were performed at each participating center using locally available methods.

Raw data were extracted from the respective instruments. Identification results with concordance percentages below 95% were excluded. The curated datasets were processed using the Backlink tool and WHONET 2025 software. Only microbiologically significant isolates obtained from lower respiratory specimens (LRS) (endotracheal aspirate and bronchoalveolar lavages; meanwhile, sputum specimens were excluded), urine, blood, biopsies and abscesses were included.

Antimicrobial susceptibility and the ESBL phenotype for *E. coli* and *K. pneumoniae* were determined using a comprehensive panel of antibiotics selected according to organism type. For each automated system, the determination of whether an organism was an ESBL producer was performed by comparing the growth inhibition of third-generation cephalosporins available in panels with and without the presence of an inhibitor (clavulanic acid).

Categories of antibiotics included selectively on panels were aminoglycosides: gentamicin (GEN), gentamicin high-dose (GEH), streptomycin high-dose (STH); carbapenems: imipenem (IPM), meropenem (MEM), ertapenem (ETP cephalosporins: cefepime (FEP), ceftazidime (CAZ), ceftriaxone (CRO), cefuroxime (CXM); cephalosporins with inhibitors: ceftazidime/avibactam (CZA), ceftolozane/tazobactam (CZT); cephamycins: cefoxitin (FOX), oxacillin (OXA); fluoroquinolones: ciprofloxacin (CIP), levofloxacin (LVX); folate pathway inhibitors: sulfamethoxazole/trimethoprim (SXT); glycopeptides: teicoplanin (TEC), and vancomycin (VAN); lincosamides: clindamycin (CLI); lipopeptides: daptomycin (DAP); monobactams: aztreonam (ATM); macrolides: erythromycin (ERY), azithromycin (AZM); nitrofuran: nitrofurantoin (NIT); oxazolidinones: linezolid (LNZ); penicillin and penicillin with inhibitors: penicillin (PEN), amoxicillin/clavulanic acid (AMC), ampicillin (AMP), ampicillin/sulbactam (SAM), piperacillin/tazobactam (TZP); streptogramins: quinupristin/dalfopristin (QDA); tetracyclines: tetracycline (TCY). Susceptibility results were categorized as susceptible, intermediate, or resistant, according to the Clinical and Laboratory Standards Institute (CLSI) M100-S35 (2025) breakpoints [8]. Some data were excluded from the tables and analysis. Specifically, results with an *n* < 30 per species/antibiotic combination, results corresponding to inactive antimicrobials against a given species, and all data related to colistin were not considered.

Among Gram-negative isolates, resistance profiles were further classified as MDR (multidrug-resistant): non-susceptibility to ≥ 1 agent in ≥ 3 antimicrobial categories; Possible XDR (extensively drug-resistant) (if data is incomplete for full XDR definition): non-susceptibility to ≥ 1 agent in all but ≤ 2 categories; XDR: full data available confirming non-susceptibility to all but ≤ 2 categories. Possible PDR (pandrug-resistant) (if data is incomplete for full PDR definition): non-susceptibility to all agents in all tested categories. The use of possible XDR or possible PDR was included if data was incomplete for the full XDR or full PDR definition; Thus, the classification was limited by the panel evaluated for each clinical isolate, in accordance with Magiorakos et al. [9]. Definitions were assigned by the WHOnet software based on the susceptibility profile and the panel of antibiotics included for each isolate. (https://whonet.org/WebDocs/Sample_Epidemiology_Report.pdf).

### Genotyping of isolates

As a result of the active surveillance by the INVIFAR network, centers sent carbapenem-resistant clinical isolates to the coordinating center for genotyping. The carbapenem resistance was confirmed by the disk diffusion method, and intermediate or resistant strains were further analyzed for genotyping. DNA was extracted by thermal lysis, and the presence of the most common carbapenemase-encoding genes was assessed by polymerase chain reaction (PCR) using previously reported primers [4]. The genes targeted in *Enterobacterales* included *bla*_NDM_, *bla*_KPC_, *bla*_VIM_, *bla*_IMP_, and *bla*_OXA-48-like_. In *Acinetobacter baumannii*, the genes screened were *bla*_NDM_, *bla*_OXA-23,_ and *bla*_OXA-24_. In *Pseudomonas aeruginosa*, the genes screened were *bla*_VIM,_ and *bla*_IMP_.

### Data analysis

Antibiotic resistance data were analyzed by species (*Escherichia coli*, *Klebsiella pneumoniae*, *Enterobacter cloacae*, *A. baumannii*, *P. aeruginosa*, *S. aureus*, and *Enterococcus faecium*), specimen type (blood, urine, LRS, abscesses, and biopsies), hospital ward (ICU, emergency departments [EME], non-ICU inpatient wards [INX], and outpatient settings [OUT]), age groups (0–18 years, 19–59 years, and ≥ 60 years), and gender (female, male). Only one isolate per patient was included in the analysis according criteria suggested by CLSI document M39 (Analysis and Presentation of Cumulative Antimicrobial Susceptibility Test Data) [10].

Frequencies of antibiotic resistance were compared using the chi-square test only if the number of clinical isolates/antibiotic was ≥ 30. Statistical analyses were performed using SPSS Statistics version 22.0 (IBM Corporation, Somers, NY, USA). A p-value of < 0.05 was considered statistically significant.

### Ethical approval

This study was approved by the Research Committee of the Instituto Nacional de Rehabilitación Luis Guillermo Ibarra Ibarra under approval number 55/22 AC. The requirement for informed consent was waived by the ethics committee. All procedures were conducted in accordance with the Declaration of Helsinki. No identifiable patient data was used.

## Results

### Participating centers and microbiological data

The study included data from 55 participating centers across 23 states in Mexico and Mexico City (Table 1). Bacterial identification methods varied by site and included: VITEK (*n* = 31), MicroScan WalkAway (*n* = 5), BD Phoenix Automated Microbiology System (*n* = 3), MALDI-TOF (*n* = 10), and Biochemical (manual) methods (*n* = 6). Antimicrobial susceptibility testing was performed using VITEK (*n* = 40), MicroScan WalkAway (*n* = 6), BD Phoenix (*n* = 4), and disk diffusion (*n* = 6) (Table 1).

**Table 1:** Characteristics of participating centers and methods used for bacterial identification and antimicrobial susceptibility testing

Center	Pu/Pr	Type of Hospital	No. Beds	ICU beds	ID method	Susceptibility method	State
Centers with hospitalization areas							
Hospital Civil “Fray Antonio Alcalde”	Pu	Gen	747	100	Vitek 2	Vitek 2	Jalisco
Hospital Universitario “Dr. José Eleuterio González”	Pu	Gen	463	22	MALDI-TOF	Vitek 2	Nuevo León
Hospital Regional General “Ignacio Zaragoza”. ISSSTE	Pu	Spe	325	12	Vitek 2	Vitek 2	Mexico City
Hospital General de Estado de Sonora	Pu	Spe	305	8	Vitek 2	Vitek 2	Sonora
Hospital General “Dr. Agustin O’Horan”	Pu	Gen	296	11	Vitek 2	Vitek 2	Yucatan
Hospital General Regional No.6 “Dr. Ignacio Garcia Tellez”	Pu	Gen	286	10	MALDI-TOF	Vitek 2	Tamaulipas
Hospital General León	Pu	Gen	260	18	BD Phoenix	BD Phoenix	Guanajuato
Hospital General “Dr. Miguel Silva”	Pu	Gen	250	16	Vitek 2	Vitek 2	Michoacán
Instituto Nacional de Rehabilitación “Luis Guillermo Ibarra Ibarra”	Pu	Spe	245	18	MALDI-TOF	Vitek 2	Mexico City
Instituto Nacional de Ciencias Médicas y Nutrición “Salvador Zubirán”	Pu	Spe	212	22	MALDI-TOF	Vitek 2	Mexico City
Instituto Nacional de Cardiología “Ignacio Chávez”	Pu	Spe	208	30	MALDI-TOF	BD Phoenix	Mexico City
Hospital Regional de Alta Especialidad Bicentenario de la Independencia	Pu	Spe	196	7	Vitek 2	Vitek 2	Mexico State
Hospital Regional de Alta Especialidad del Bajio	Pu	Spe	184	16	Vitek 2	Vitek 2	Guanajuato
Hospital General de Zona No.1 IMSS Nueva Frontera	Pu	Gen	180	28	Vitek 2	Vitek 2	Chiapas
Hospital General de Zona No. 35	Pu	Gen	180	6	Vitek 2	Vitek 2	Chihuahua
Hospital Chiapas Nos Une. “ Dr. Jesus Gilberto Gomez Maza”.	Pu	Gen	180	20	Vitek 2	Vitek 2	Chiapas.
Centro Médico “Dr. Ignacio Chávez”	Pu	Gen	174	4	Vitek 2	Vitek 2	Sonora
Hospital General de Querétaro	Pu	Gen	169	9	Vitek 2	Vitek 2	Querétaro
Hospital de la Niñez Poblana	Pu	Ped	164	9	Vitek 2	Vitek 2	Puebla
Instituto Nacional de Enfermedades Respiratorias “Ismael Cosio Villegas”	Pu	Spe	161	15	MALDI-TOF	Vitek 2	Mexico City
Hospital General “Juan María de Salvatierra”	Pu	Gen	157	18	Vitek 2	Vitek 2	Baja California Sur
Hospital General y Hospital del niño y la mujer (IMSS Bienestar)	Pu	Gen	156	6	Vitek 2	Vitek 2	Sonora
Instituto Nacional de Cancerologia	Pu	Spe	150	8	MALDI-TOF	Vitek 2	Mexico City
Hospital Regional “Alta Especialidad” ISSSTE Monterrey	Pu	Spe	150	10	MicroScan	MicroScan	Nuevo León
Centenario Hospital Miguel Hidalgo	Pu	Spe	144	30	MALDI-TOF	Vitek 2	Aguascalientes
Hospital General de Zona No. 21	Pu	Gen	139	9	MicroScan	MicroScan	Jalisco
Hospital “Dr Manuel GEA Gonzalez”	Pu	Gen	130	10	MALDI-TOF	Vitek 2	Mexico City
Hospital Infantil de Morelia	Pu	Ped	130	18	Vitek 2	Vitek 2	Michoacán
Hospital General Silao	Pu	Gen	118	14	BD Phoenix	BD Phoenix	Guanajuato
Hospital para el Niño Toluca IMIEM	Pu	Spe	116	7	Vitek 2	Vitek 2	Mexico State
HGZ #46 IMSS Tabasco	Pu	Gen	112	6	Vitek 2	Vitek 2	Tabasco
Hospital General ISSSTE San Luis Potosí	Pu	Gen	111	4	MicroScan	MicroScan	San Luis Potosí
Hospital General “Lázaro Cárdenas”	Pu	Gen	110	6	MicroScan	MicroScan	Chihuahua
HG “Dr. Fernando Ocaranza”	Pu	Gen	105	4	Vitek 2	Vitek 2	Sonora
Hospital de Especialidades Pediátricas IMSS-Bienestar	Pu	Ped	90	24	Vitek 2	Vitek 2	Chiapas
Hospital Ángeles Morelia	Pr	Spe	67	12	Vitek 2	Vitek 2	Michoacán
Hospital Regional de Alta Especialidad de Oaxaca	Pu	Spe	66	10	Vitek 2	Vitek 2	Oaxaca
Hospital del niño “Dr. Federico Gómez Santos”	Pu	Ped	44	13	Vitek 2	Vitek 2	Coahuila
Hospital Español Veracruz	Pr	Gen	40	6	Vitek 2	Vitek 2	Veracruz
Hospital del Niño Morelense	Pu	Ped	35	2	Vitek 2	Vitek 2	Morelos
Hospital “Adolfo López Mateos”-ISSSTESON	Pu	Gen	33	22	Vitek 2	Vitek 2	Sonora
Departamento de Microbiologia del Hospital la Luz	Pr	Gen	28	3	Biochemical (manual)	Disk diffusion	Michoacán
Centers with no hospitalization area							
Lapi Laboratorio Médico	Pr	NA	NA	NA	Vitek 2	Vitek 2	Mexico City
Laboratorio Dorado	Pr	NA	NA	NA	MALDI-TOF	Vitek 2/MicroScan	Baja California
Laboratorio Clinico y Bacteriologico LB	Pr	NA	NA	NA	Biochemical (manual)	Disk diffusion	Jalisco
Bio Diagnostics Laboratorio Clínico S.C.	Pr	NA	NA	NA	Vitek 2	Vitek 2	Tlaxcala
Laboratorio SEMLAB	Pr	NA	NA	NA	Biochemical (manual)	Disk diffusion	Michoacán
Laboratorio Futura Medica	Pr	NA	NA	NA	Biochemical (manual)	Disk diffusion	Michoacán
Centro Integral de Atención a la Salud Unidad Sur ISSSTESON	Pu	NA	NA	NA	Vitek 2	Vitek 2	Sonora
Laboratorios del Centro	Pr	NA	NA	NA	Vitek 2	Vitek 2	Michoacán
Diagnóstico Médico Integral “Pasteur”	Pr	NA	NA	NA	BD Phoenix	BD Phoenix	Mexico State
Laboratorio BIOCLIN	Pr	NA	NA	NA	Vitek 2	Vitek 2	Guerrero
Laboratorio DIPROMI	Pr	NA	NA	NA	Microscan	Microscan	Michoacán
Centro Universitario de Salud, Universidad Autónoma de Nuevo León.	Pu	NA	NA	NA	Biochemical (manual)	Disk diffusion	Nuevo León
Centro de diagnóstico microbiológico SA de CV	Pr	NA	NA	NA	Biochemical (manual)	Disk diffusion	Michoacán

Pr: Private, Pu: Public, NA: Not Applicable, ID: identification

General: Gen, Pediatric: ped, ICU: intensive care unit

A total of 11,290 clinical isolates were included in the analysis. The majority were obtained from urine (*n* = 7,149; 63.3%), followed by blood (*n* = 1,370; 12.1%), LRS (*n* = 1,317; 11.7%), biopsies (*n* = 886; 7.8%), and abscesses (*n* = 568; 5.0%). Of all isolates, 9,345 (82.8%) were Gram-negative bacteria, including 7,853 *Enterobacterales* (69.6%) and 1,492 non-fermenting Gram-negative rods (13.2%). The remaining 1,945 isolates (17.2%) were Gram-positive cocci.

*E. coli* was the most frequently isolated pathogen overall (*n* = 6,185; 54.8%), followed by *K. pneumoniae* (*n* = 1,365; 12.1%) and *P. aeruginosa* (*n* = 1,110; 9.8%). *E. coli* was also the most common pathogen isolated from urine (*n* = 5,109; 71.5%), blood (*n* = 466; 34.0%), biopsies (*n* = 239; 27.0%), and abscesses (*n* = 199; 35.0%). In LRS, *P. aeruginosa* was the most frequently recovered pathogen (*n* = 392; 29.8%), followed by *S. aureus* (*n* = 303; 23.0%).

Most of *E. coli* (*n* = 3,766; 57.4%), *K. pneumoniae* (*n* = 752; 50.4%), and *P. aeruginosa* (*n* = 383; 28.9%) isolates were classified as MDR (Table 2); however, *A. baummannii* isolates had the highest rate of XDR (*n* = 65; 15.4%) and possible PDR isolates (*n* = 202; 47.9%).

**Table 2:** Distribution of multidrug-resistant (MDR), possible extensively drug-resistant (possible XDR), extensively drug-resistant (XDR), and possible pandrug-resistant (possible PDR) isolates among Gram-negative bacteria

	MDR *n* (%)	Possible XDR *n* (%)	XDR *n* (%)	Possible PDR *n* (%)
*E. coli* (*n* = 6560)	3766 (57.4)	1323 (20.2)	0 (0.0)	7 (0.1)
*K. pneumoniae* (*n* = 1,493)	752 (50.4)	506 (33.9)	0 (0.0)	17 (1.1)
*A. baumannii* (*n* = 422)	289 (68.5)	216 (51.2)	65 (15.4)	202 (47.9)
*P. aeruginosa* (*n* = 1,324)	383 (28.9)	312 (23.6)	42 (3.2)	162 (12.2)

### Drug resistance in Gram-negative bacteria by age group

**Fig. 1:**
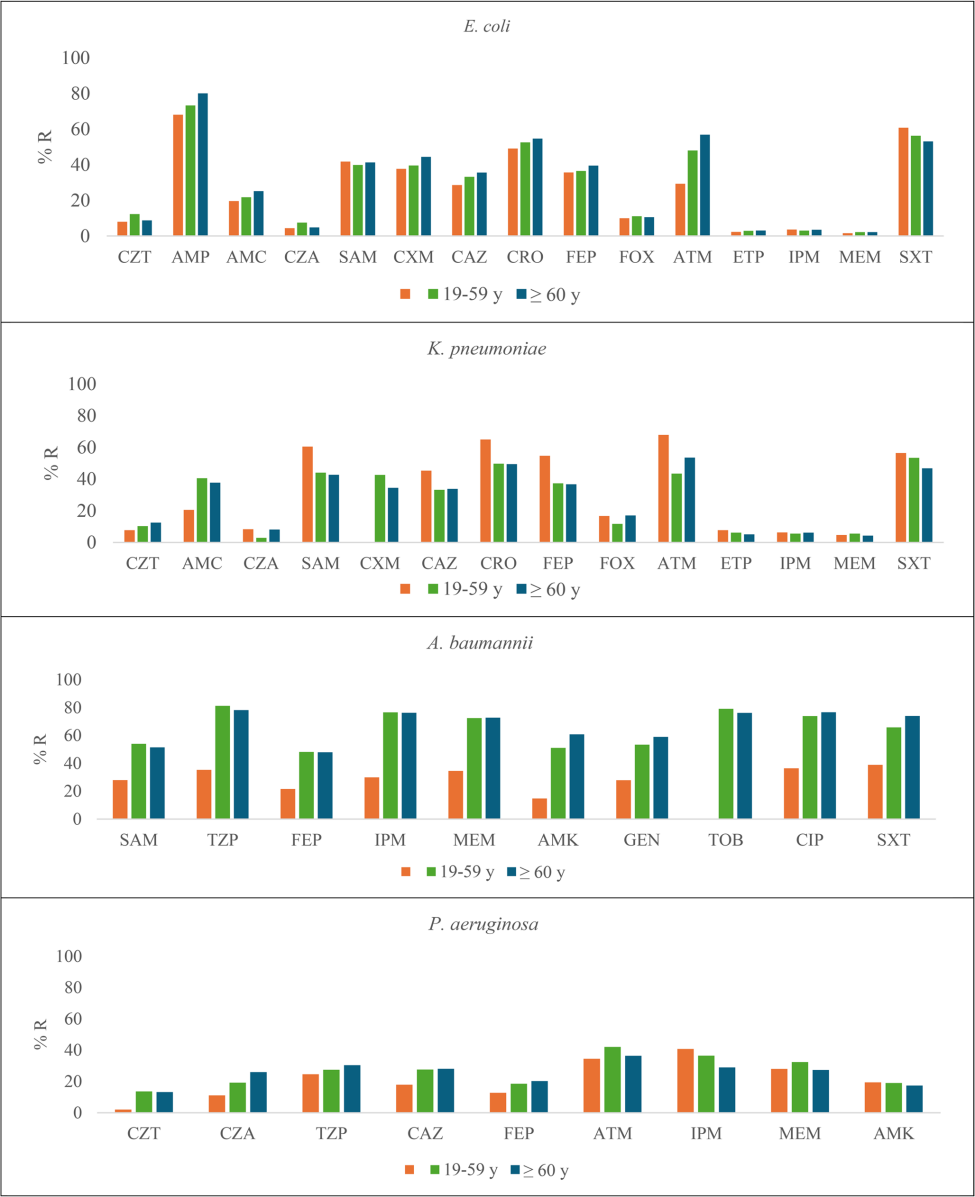
Distribution of Antibiotic resistance among age groups. CZT: Ceftolozane/Tazobactam, AMP: Ampicillin, AMC: Amoxicillin/Clavulanic acid, CZA: Ceftazidime/Avibactam, SAM: Ampicillin/Sulbactam, CXM: Cefuroxime, CAZ: Ceftazidime, CRO: Ceftriaxone, FEP: Cefepime, FOX: Cefoxitin, ATM: Aztreonam, ETP: Ertapenem, IPM: Imipenem, MEM: Meropenem, AMK: Amikacin, GEN: Gentamicin, CIP: Ciprofloxacin, LVX: Levofloxacin, SXT: Sulfamethoxazole/Trimethoprim, TGC: Tigecycline, TZP: Piperacillin/Tazobactam, TOB: Tobramycin

In the > 60 years age group, *E. coli* showed higher resistance rates to AMP (80.1%, *p* < 0.013), CAZ (35.6%, *p* = 0.008), and ATM (56.9%, *p* < 0.001) than the other two age groups; however in the 0–18 years group a higher resistance rate to SXT (60.8%, *p* = 0.007) was observed. Extended spectrum ß-lactamases (ESBL)-producing isolates were similarly distributed among age groups (*p* = 0.264) (Fig. 1, Suppl. Tables 1 and Suppl Table 2).

For *K. pneumoniae*, higher resistance rates were observed in the 0–18 years group for SAM (60.5%, *p* < 0.001), CAZ (45.3%, *p* = 0.010), CRO (65%, *p* = 0.001), and FEP (54.7%, *p* < 0.001). Moreover, the highest proportion of ESBL-producer isolates was detected in this species in the 0–18 years group (65.9%, *p* = 0.001) (Fig. 1, Suppl. Tables 1 and Suppl Table 2).

For *A. baumannii*, higher resistance rates were observed in the 19–59 years group for SAM (54.0%, *p* = 0.004), TZP (81.3%, *p* < 0.001), FEP (48.2%, *p* = 0.002), and IPM (76.6%, *p* < 0.001). In the > 60 years, the higher resistance rates were for MEM (72.8%, *p* < 0.001), AMK (60.8%, *p* < 0.001), and GEN (59.0%, *p* = 0.002) than the other age groups. Among *P. aeruginosa* isolates, resistance to CZA (26.0%, *p* = 0.043), and CAZ (28.1%, *p* = 0.024) was higher in the > 60 years group than the other age groups; while resistance to IPM was higher in the 0–18 years group (40.8%, *p* = 0.019) than the other age groups (Fig. 1, Suppl. Table 2).

### Drug resistance in Gram-negative bacteria according to the facility

**Fig. 2:**
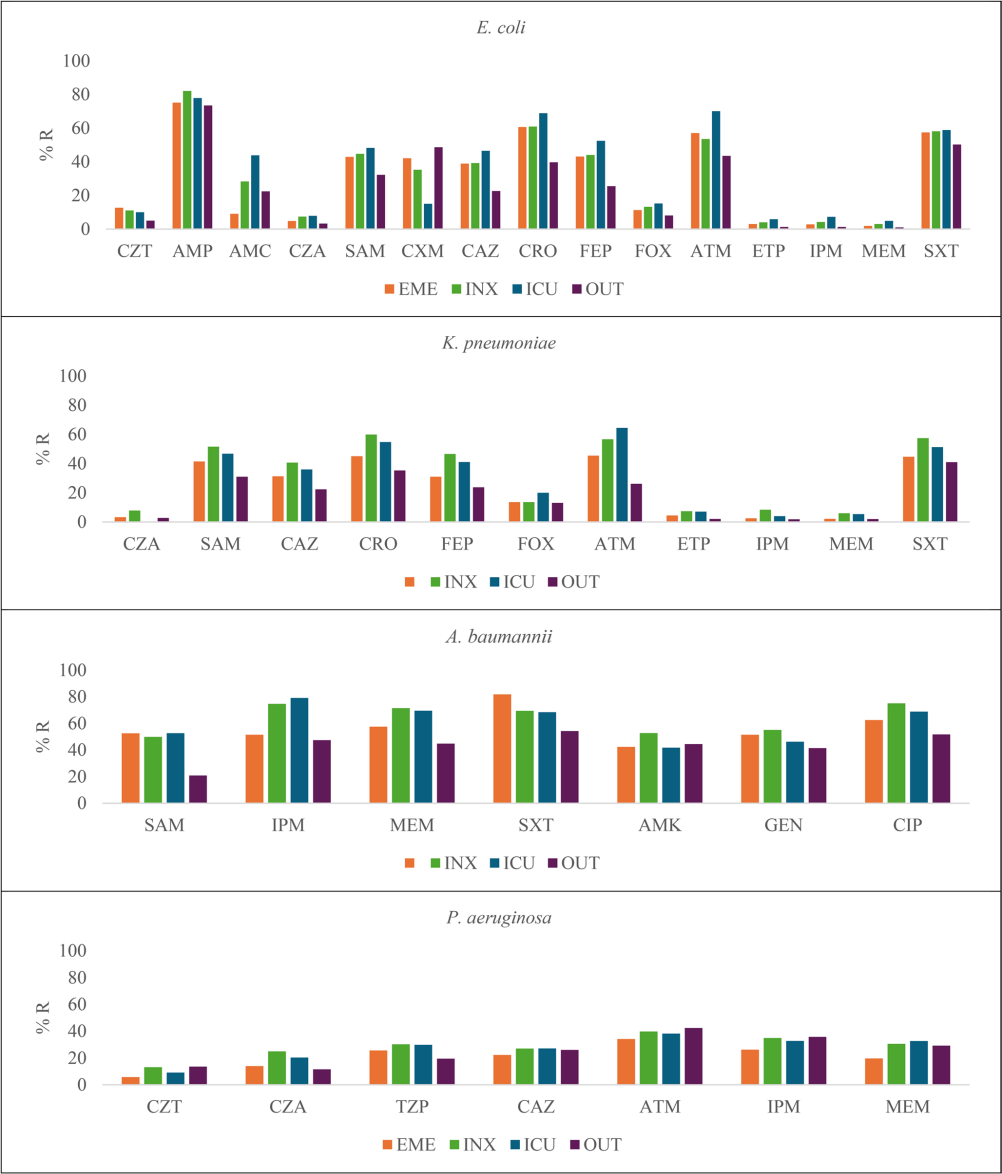
Distribution of Antibiotic resistance according to sites of care. CZT: Ceftolozane/Tazobactam, AMP: Ampicillin, AMC: Amoxicillin/Clavulanic acid, CZA: Ceftazidime/Avibactam, SAM: Ampicillin/Sulbactam, CXM: Cefuroxime, CAZ: Ceftazidime, CRO: Ceftriaxone, FEP: Cefepime, FOX: Cefoxitin, ATM: Aztreonam, ETP: Ertapenem, IPM: Imipenem, MEM: Meropenem, AMK: Amikacin, GEN: Gentamicin, CIP: Ciprofloxacin, LVX: Levofloxacin, SXT: Sulfamethoxazole/Trimethoprim, TGC: Tigecycline, TZP: Piperacillin/Tazobactam, TOB: Tobramycin. EME: emergency department, INX: non-ICU inpatient wards, ICU: intensive care unit, and OUT: outpatient settings

Most *E. coli* isolates had the highest resistance in the ICU area, with high rates resistance to CAZ (46.5%, *p* < 0.001), CRO (68.8%, *p* < 0.001), FEP (52.4%, *p* < 0.001), ATM (70%, *p* = 0.002), ETP (5.9%, *p* < 0.001), IPM (7.3%, *p* < 0.001), MEM (4.9%, *p* < 0.001), and SXT (58.8%, *p* < 0.001). However, the highest proportion of ESBL producers isolated was reported from the EME area (57.7%, *p* < 0.001) (Fig. 2, Suppl. Tables 1 and 3).

On the contrary, *K. pneumoniae* isolates had the highest resistance in the INX area, with high rates resistance to AMC (40%, *p* < 0.033), SAM (51.6%, *p* < 0.001), CAZ (40.7%, *p* < 0.001), CRO (59.9%, *p* < 0.001), FEP(46.6%, *p* < 0.001), ETP (7.4%, *p* = 0.004), IPM (8.4%, *p* = 0.025), and SXT (57.5%, *p* < 0.001); similarly, the highest proportion of ESBL-producer isolates were reported at this area (57.7%, *p* < 0.001) (Fig. 2, Suppl. Table 3).

For *A. baumannii*, higher resistance rates were observed in the ICU area for SAM (52.6%, *p* = 0.039), and IPM (79.1%, *p* < 0.002), and in the INX area for MEM (71.4%, *p* < 0.015), and CIP (75.1%, *p* < 0.033). Among *P. aeruginosa* isolates, CZA resistance was higher (24.9%, *p* = 0.046) in the INX area, while resistance to MEM was higher in the ICU ward (32.6%, *p* = 0.040) (Fig. 2, Suppl. Table 3).

### Drug resistance in Gram-negative bacteria by specimen

**Fig. 3:**
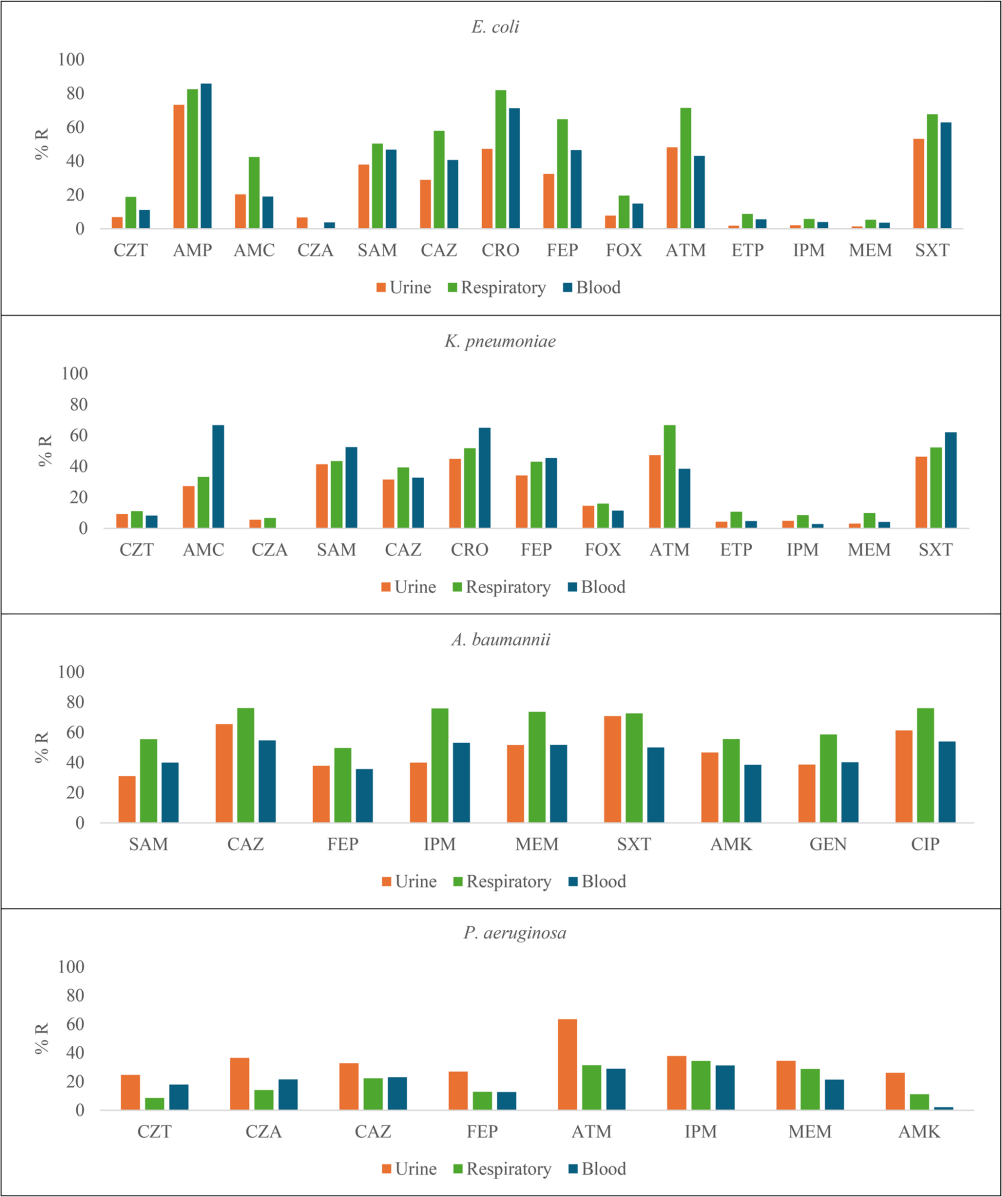
Distribution of Antibiotic resistance according to clinical specimen. CZT: Ceftolozane/Tazobactam, AMP: Ampicillin, AMC: Amoxicillin/Clavulanic acid, CZA: Ceftazidime/Avibactam, SAM: Ampicillin/Sulbactam, CAZ: Ceftazidime, CRO: Ceftriaxone, FEP: Cefepime, FOX: Cefoxitin, ATM: Aztreonam, ETP: Ertapenem, IPM: Imipenem, MEM: Meropenem, AMK: Amikacin, GEN: Gentamicin, CIP: Ciprofloxacin, LVX: Levofloxacin, SXT: Sulfamethoxazole/Trimethoprim, TZP: Piperacillin/Tazobactam

*E. coli* respiratory isolates had the highest rates of resistance to CAZ (57.8%, *p* < 0.001), FEP (64.7%, *p* < 0.001), FOX (19.6%, *p* = 0.008), ATM (71.4%, *p* = 0.008), ETP (8.8%, *p* < 0.001), IPM (5.8%, *p* = 0.016), MEM (5.3%, *p* < 0.001), and SXT (67.6%, p = *p* < 0.001), along with the highest proportion of ESBL-producers (75.9%, *p* < 0.001) (Fig. 3, Suppl. Tables 1 and Suppl. Table 4). A high proportion of resistance (> 60%) to CRO, CIP, and SXT was observed among *E. coli* isolates from biopsies, as well as a high proportion of ESBL-producer isolates was detected in both biopsies (68.8%) and abscesses (68.2%) (Suppl Table 5).

On the contrary, *K. pneumoniae* had the highest resistance percentage in blood isolates, with elevated rates of resistance to SAM (52.5%, *p* = 0.028) and SXT (62.1%, *p* = 0.014). Similarly, most ESBL-producers were reported from this specimen (63.2%, *p* = 0.001). Among respiratory isolates, resistance percentages were higher for ATM (66.7%, *p* = 0.034), ETP (10.8%, *p* = 0.001), and MEM (9.9%, *p* < 0.001) than the other clinical specimens (Fig. 3, Suppl. Table 4). A high proportion of resistance to SXT (68.4%) was observed in *K. pneumoniae* isolates from biopsies, along with a high proportion of ESBL production in isolates from both biopsies (65.6%) and abscesses (42.9% (Suppl Tables 1 and 5).

For *A. baumannii*, higher resistance proportion were observed among respiratory isolates, to SAM (55.5%, *p* = 0.009), TZP (77.1%, *p* = 0.029), CAZ (76.1%, *p* = 0.002), MEM (73.6%, *p* < 0.001), GEN (58.6%, *p* = 0.009), and CIP (76%, *p* = 0.001) (Fig. 3, Suppl. Table 4). Also, there was a high proportion of resistance to TZP (94.7%), CAZ (92.9%), IPM (94.7%), MEM (92.9%), and CIP (95.2%) in biopsy isolates (Suppl Table 5).

Among *P. aeruginosa*, resistance was higher among urine isolates, to CZT (24.7%, *p* = 0.008), CZA (36.6%, *p* < 0.001), ATM (63.6%, *p* < 0.001), MEM (34.5%, *p* = 0.009), and AMK (26.1%, *p* < 0.001) (Fig. 3, Suppl. Table 4). Moreover, there was a high proportion of resistance to IMP in both isolates from biopsies (38.2%) and abscesses (37.8%); with comparable results observed for MEM (34.7% and 45.9%, respectively) (Suppl. Table 5).

### Drug resistance in Gram-negative bacteria by gender

Drug resistance in *E. coli* and *K. pneumoniae* was higher in the male group to all antibiotics evaluated (except for CZA, FOX, ATM, and IPM in *K. pneumoniae* isolates). There were no significant differences in drug resistance for *A. baumannii* by gender; only resistance to CZT and AMK was higher in female and male groups, respectively (Suppl Table 8).

### Drug resistance in Gram-positive bacteria

**Fig. 4:**
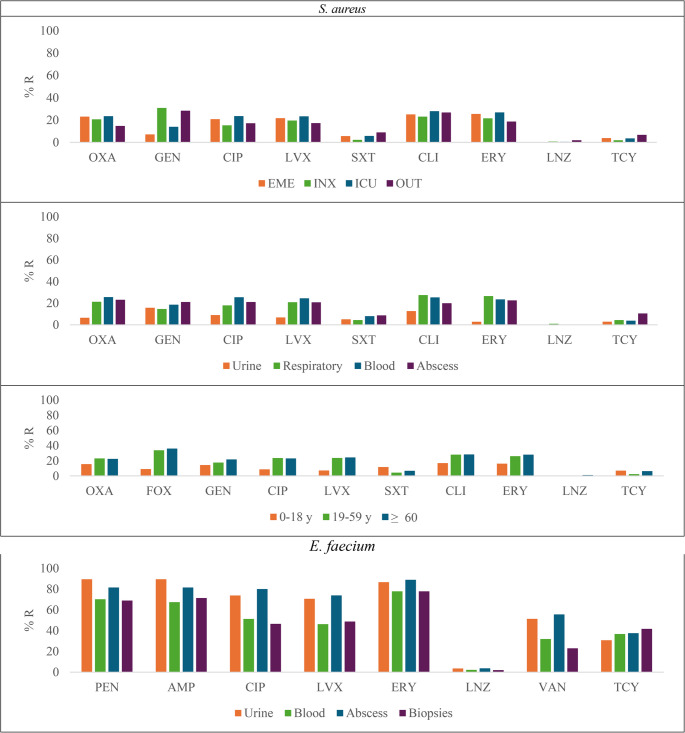
Distribution of Antibiotic resistance according to site of care, clinical specimen and age group for *S. aureus* and according to clinical specimen for *E. faecium*. OXA: Oxacillin, FOX: Cefoxitin, GEN: Gentamicin, CIP: Ciprofloxacin, LVX: Levofloxacin, SXT: Sulfamethoxazole/Trimethoprim, CLI: Clindamycin, ERY: Erythromycin, LNZ: Linezolid, VAN: Vancomycin, TCY: Tetracycline, PEN: Penicillin, AMP: Ampicillin. EME: emergency departments, INX: non-ICU inpatient, ICU: intensive care units, and OUT: outpatient settings

There were no significant differences in resistance rates among *S. aureus* isolates when analyzed by ward. However, *S. aureus* recovered from blood showed the highest resistance to OXA (25.7%, *p* < 0.004), CIP (25.5%, *p* < 0.004), and LVX (24.5%, *p* = 0.011). Also, isolates from LRS showed higher resistance percentages to CLI (27.5%, *p* = 0.040) and ERY (26.6%, *p* < 0.001). Finally, among all age groups, the > 60 years group exhibited the highest resistance rates of *S. aureus* to LVX (24.4%, *p* < 0.001), CLI (28.3%, *p* = 0.020) and ERY (28%, *p* = 0.022) (Fig. 4, Suppl. Table 4). There were no differences in antibiotic resistance when *S. aureus* isolates were analyzed by gender (Suppl. Table 8). For *E. faecium*, the highest resistance rates to CIP (80%, *p* < 0.001), LVX (73.9%, *p* = 0.010), and VAN (55.6%, *p* < 0.001) were detected in abscess isolates (Fig. 4, Supp Table 7).

### Distribution of carbapenemase-encoding genes

Carbapenem-resistant isolates, including *E. coli* (*n* = 56), *K. pneumoniae* (*n* = 17), *E. cloacae* (*n* = 7), *A. baumannii* (*n* = 108), and *P. aeruginosa* (*n* = 108), were received by the coordinating center for genotyping. All received clinical isolates were processed, and the results are included in this manuscript.

Among *E. coli* isolates, the most frequent carbapenemase-encoding gene was *bla*_OXA-48-like_ (26/56, 46.4%), followed by *bla*_NDM_ (24/56, 42.9%); for *K. pneumoniae* isolates, the most frequent carbapenemase-encoding gene was *bla*_NDM_ (7/17, 41.2%), followed by bla_OXA-48-like_ (6/17, 35.3%) (Table 3). Four isolates of *E. cloacae* carried *bla*_NDM,_ and one isolate carried *bla*_NDM_ and *bla*_VIM_.

**Table 3:** Distribution of carbapenemase-encoding genes in *Enterobacterales*

*n*	MEM	PCR result
*E. coli* (*n* = 56)		
20	R	*bla*_NDM_
17	R	*bla*_OXA−48−like_
6	I	*bla*_OXA−48−like_
5	R	All negative
2	R	*bla*_N__DM,_ *bla*_OXA−48−like_
1	R	*bla*_KPC_
1	R	*bla*_NDM_, *bla*_VIM_
1	R	*bla*_VIM_, *bla*_OXA−48−like_
1	I	All negative
1	I	*bla*_NDM_
1	I	*bla*_VIM_
*K. pneumoniae* (*n* = 17)		
6	R	*bla*_NDM_
4	R	*bla*_OXA−48−like_
3	R	All negative
1	R	*bla*_NDM,_ *bla*_OXA−48−like_
1	R	*bla*_VIM_
1	R	*bla*_KPC,_ *bla*_OXA−48−like_
1	I	*bla*_VIM_
*E. cloacae* (*n* = 7)		
4	R	*bla*_NDM_
1	R	All negative
1	R	*bla*_NDM,_ *bla*_VIM_
1	I	All negative

*R* resistant, *I* intermediate, *MEM* meropenem

In *A. baumannii* isolates, the most frequent carbapenemase-encoding gene was *bla*_OXA-24_ (79/108, 73.1%), followed by *bla*_OXA-23_ (21/108, 19.4%); among *P. aeruginosa* isolates, the most frequent carbapenemase-encoding gene was *bla*_IMP_ (18/108, 16.7%) (Table 4).

**Table 4:** Distribution of carbapenemase encoding genes in *A. baumannii* and *P. aeruginosa*

*n*	Meropenem	PCR result
*A. baumannii* (*n* = 108) *		
79	R	*bla*_OXA24_
21	R	*bla*_OXA23_
7	R	All negative
1	I	All negative
*P. aeruginosa* (*n* = 108)		
78	R	All negative
17	R	*bla*_IMP_
8	R	*bla*_VIM_
2	I	All negative
1	R	*bla*_IMP_
1	I	All negative
1	R	*bla*_VIM_

All *A. baumannii* isolates were evaluated for *bla*_OXA23_ and *bla*_OXA24,_ and *bla*_NDM_

All clinical isolates of *P. aeruginosa* were evaluated for *bla*_VIM_ and *bla*_IMP_

R: resistant, I: intermediate

## Discussion

AMR is a public health and economic concern due to its critical impact on human health, specially by the rapid global spread of resistant bacteria to multiple or all treatment options [1]. This study aimed to report the distribution of AMR among some WHO high-priority pathogens during the first quarter of 2025, alongside the detection of carbapenemase-encoding genes in strains submitted by members of the INVIFAR network, and detected that the most frequently recovered pathogen in the included clinical specimens was *E. coli* (nearly 55%) followed by *K. pneumoniae* (nearly 12%) According to the WHO Bacterial Priority Pathogens list, *E. coli* and *K. pneumoniae* are among the most frequently recovered microorganisms [7]. Reflecting its importance, the WHO established a tricycle program to oversee the surveillance of *E. coli* from one health perspective, with a particular focus on ESBL-producer strains [11]. In our study, no statistically significant differences were observed in ESBL-producers according to the age groups analyzed; however, ESBL frequency was high (from 52 to 54.4%). Comparatively, the SMART surveillance program reported an ESBL frequency in *E. coli* between 13.4 and 19.2% in the United States, 28.4% to 45.5% in Latin America, and nearly 65% in Mexico from 2015 to 2019 [12], which is consistent with our findings.

For *K. pneumoniae*, the prevalence of ESBL-producers has been reported to range from 28% in India [13] to 84% in Ghana [14]; In both studies, data were derived from single hospitals. Our findings (~ 60%) align with those reported in our 2024 report [4].

Among pediatric patients (0–18 y), 65.9% of *K. pneumoniae* were ESBL producers, similar to previous findings from our network (68%) [15]. Similar results have been reported for a population from Poland, with a frequency of *K. pneumoniae* ESBL producers of 80% [16].

*E. coli* is the predominant pathogen in both uncomplicated and complicated urinary tract infections, accounting for over 60% of cases [17, 18] and bloodstream infections [19]. Although clinical data confirming the isolated pathogens as causative agents, especially for urine, was not included in our study, our findings are consistent with these reports, showing 71.5% of *E. coli* strains recovered from urine and 34% from blood.

In LRS, the most frequently reported species was *P. aeruginosa*, which is associated with both community and hospital-acquired infections, particularly in immunocompromised individuals such as patients with neutropenia, burns, cancer, organ transplant, and ICU patients [20]. While we lacked detailed data for the clinical condition of patients, we did observe higher resistance levels in *P. aeruginosa* for ICU patients. Resistance dynamics vary across hospital wards due to differences in antimicrobial pressure and patient complexity. In the ICU, high resistance rates reflect selective pressure and prolonged hospital stays. In EME, which interfaces with the community, resistance patterns may reflect broader community trends [21].

In this study, *A. baumannii* was frequently isolated from LRS (13.7%). A previous study reported a higher frequency of resistance to IPM in LRS isolates (> 70%) compared to those recovered from blood (30%), urine (50%), and soft tissue samples (60%) [22]. Regarding *A. baumannii*, the XDR isolates were 15.4% (in isolates recovered from all clinical specimens included in this study), and this percentage is lower than the value observed in patients hospitalized in the ICU, in Jordan (90%) [23], and Iran (59.5%) [24].

Regarding Gram-positive, 25.7% of *S. aureus* strains were resistant to OXA, slightly above the 21.8% of the previous report [25]. *S. aureus* is the most frequently reported antibiotic-resistant pathogen and remains a major contributor to morbidity and mortality [26].

The need for surveillance stems from its critical role in preserving antimicrobial efficacy, especially as the pipeline for new molecules is limited. Alternative strategies include combining existing agents with ß-lactamase inhibitors, such as CZA [27], CZT [28] or meropenem-vaborbactam [29] which target key resistance mechanisms and are valuable alternatives for some MDR organisms. Regarding these new combinations, resistance to CZA reached 26% in patients ≥ 60 years for *P. aeruginosa*. Importantly, resistance rates above 20% need to be evaluated for empirical therapies underlying the need for performing laboratory susceptibility tests [30, 31]; moreover, in our 2024 report, we detected higher resistance for CZA (23%) in the age group 18–59 years and 23.6% in the > 60 years age group [4].

Earlier studies in Mexico showed a low frequency of carbapenem resistance in *Enterobacterales*, with MEM resistance nearly 3% in *E. coli* and 12.5% in *K. pneumoniae* [32]. However, an increase in carbapenem resistance was reported after pandemics, increasing to 21.4% in *K. pneumoniae* recovered from blood [33]. In our previous study, blood isolates of *K. pneumoniae* had a 5.6% resistance rate to IPM and an 8.4% resistance rate to ETP and MEM; furthermore, *E. coli* had a resistance rate of 2.4% to MEM, 3.5% to ETP, and 3.6% to IPM [4].

Among *P. aeruginosa* isolates, resistance was higher to MEM in urine isolates (34.5%, *p* = 0.0089), with similar data reported in Turkey for MEM (30.1%) and IPM (28.0%) in a metanalysis of 10 years [34]. These data are much higher than a report from Spain, where lower resistance rates were observed with < 10% to MEM and IPM [35]. These data may reflect differences in antibiotic control strategies among countries.

Since 2021, the INVIFAR network has conducted surveillance of carbapenemase-encoding genes in selected *Enterobacterales* species. In the initial report from 2021, *bla*_NDM-1_ was the most frequently detected encoding gene found in 60% of *E. coli* (9/15) and 100% of *K. pneumoniae* (4/4) isolates collected over a three-month period [36]. Following the COVID-19 pandemic, the reported frequencies increased significantly. In 2022, *bla*_NDM-1_ was identified in 90% (72/80) of carbapenem-resistant *E. coli* and 83.5% (86/103) of *K. pneumoniae* isolates collected over an eight-month period [37]. In 2023, INVIFAR surveillance detected *bla*_NDM_ in 84% (63/75) of meropenem-resistant *E. coli* and 50% (22/44) of *K. pneumoniae* isolates, also collected over a three-month period [5]. The INVIFAR 2024 analysis showed *bla*_NDM-1_ in 64% (16/25) of meropenem-resistant *E. coli* isolates and was not detected in *K. pneumoniae.* These isolates were collected over a six-month period [4]. According to current INVIFAR surveillance data from 2025, the frequency of *bla*_NDM-1_ has a trend to diminish for *E. coli*: it was detected in 42.9% (24/56) of *E. coli* and 41.2% (7/17) of *K. pneumoniae* clinical isolates collected over a three-month period. This downward trend may suggest changes in selective pressures, infection control practices, or clonal dynamics in healthcare settings.

The distribution of carbapenemase-encoding genes in *Enterobacterales* varies worldwide. For example, a study from ICUs in Italy reported that 77 carbapenem-resistant isolates (81.9%) carried *bla*_KPC_ [38, 39] while in India, *bla*_OXA-48-like_ was detected in 37 *E. coli* isolates (20.7%) [39]. This gene is endemic in parts of Europe, North Africa, and the Middle East, and is often underdetected in non-endemic areas due to diagnostic challenges [39–41].

In *P. aeruginosa* isolates, the most frequent gene was *bla*_IMP_ (18/108, 16.7%). In contrast, a study in Italy found *bla*_VIM-2_ (5/52 9.6%) as the predominant gene in Italy [38]. The global spread of carbapenemase-encoding genes correlates with regional antimicrobial use patterns, and clone expansion is linked with the current endemic carbapenemase encoding gene and the carbapenem usage in hospitals; *bla*_KPC_ in the United States, *bla*_NDM_ in India, and particularly *bla*_IMP-6_ in South Korea [42], which supports that the AMR frequencies are not generalizable to the national and regional levels.

Gender differences can influence diagnosis and treatment practices. Women are 27% more likely than men to receive antibiotics [43]; on the other hand, in male-dominated professions, such as animal husbandry, industrial agriculture, and slaughterhouses, there is an increased exposure to antibiotics and drug-resistant pathogens [43]. The demanding nature of their jobs often delays medical care, leading to delayed diagnoses. Males are 1.5 times more likely to develop infections and have higher mortality rates from antimicrobial-resistant bacteria [43–45]. Our data confirmed higher resistance rates in *E. coli* and *K. pneumoniae* isolates from males, except CZA, FOX, ATM, and IPM in *K. pneumoniae*.

Certainly, our study has some limitations; First, methodological heterogeneity across centers in bacterial identification and susceptibility testing; Second, the possible underrepresentation of some regions or healthcare levels; third, despite WHONET data curation and centralized validation, local laboratory differences could have affected resistance categorization; Fourth, incomplete susceptibility data and variation in the antibiotic panels used across centers limited comparability and fifth that molecular analyses were restricted to carbapenem-resistant isolates, and genomic context (e.g., plasmid typing, whole-genome sequencing) were not explored for this work.

## Conclusions

This surveillance study underscores the high and heterogeneous burden of AMR in Mexico, with elevated levels of multidrug resistance, ESBL-producers, and carbapenemase activity among priority pathogens, including some *Enterobacterales*, *P. aeruginosa*, and *A. baumannii.* Age group, clinical setting, specimen type, and pathogen-specific patterns significantly influenced resistance rates, highlighting the complexity of AMR dynamics across the country The results of this study underscore the need to improve public health interventions such as epidemiologic surveillance and antibiotic stewardship program implementation in hospitals in Mexico; moreover, this report represents a continuous prospective surveillance of AMR in Mexico. This study confirms a high burden of AMR among priority pathogens in Mexico and highlights the urgent need for reinforced public health strategies, including enhanced molecular surveillance, rapid diagnostics, and antimicrobial stewardship.

## Supplementary Information

Below is the link to the electronic supplementary material.

Supplementary Material 1

Supplementary Material 2

Supplementary Material 3

Supplementary Material 4

Supplementary Material 5

Supplementary Material 6

Supplementary Material 7

Supplementary Material 8

## Data Availability

No datasets were generated or analysed during the current study.
