# Supplementary material for: The threat of multidrug-resistant microorganisms: active surveillance of key antimicrobial resistant pathogens in 2025 - a report from the INVIFAR network
Source: Eur J Clin Microbiol Infect Dis. 2026 Jan 6;45(4):1041–57. doi: 10.1007/s10096-025-05330-2 (PMC13086762; doi:10.1007/s10096-025-05330-2)
Supplement: Supplementary file 1 — Supplementary Material 1 [file 10096_2025_5330_MOESM1_ESM.docx]

Suppl Table 1. Percentage of Extended spectrum β-lactamases in *E. coli* and *K. pneumoniae* according to study groups.

| **Group** | **0-18 y** | **19-59 y** | **≥ 60 y** |
| --- | --- | --- | --- |
| *E. coli* (%) | 53.9 | 52.0 | 54.4 |
| *K. pneumoniae* (%) | 65.9 | 48.1 | 53.2 |
| **Group** | **Emergency** | **Hospitalized non-intensive care unit (ICU)** | **ICU** |
| *E. coli* (%) | 57.7 | 56.9 | 68 |
| *K. pneumoniae* (%) | 48.2 | 57.5 | 54 |
| **Group** | **Urine** | **Respiratory** | **Blood** |
| *E. coli* (%) | 47 | 75.9 | 60.6 |
| *K. pneumoniae* (%) | 47.2 | 47.4 | 63.2 |
