# Supplementary material for: The threat of multidrug-resistant microorganisms: active surveillance of key antimicrobial resistant pathogens in 2025 - a report from the INVIFAR network
Source: Eur J Clin Microbiol Infect Dis. 2026 Jan 6;45(4):1041–57. doi: 10.1007/s10096-025-05330-2 (PMC13086762; doi:10.1007/s10096-025-05330-2)
Supplement: Supplementary file 2 — Supplementary Material 2 [file 10096_2025_5330_MOESM2_ESM.docx]

Suppl Table 2. Distribution of antibiotic resistance in Gram negatives according to age groups.

|  | 0-18 y | | | |  | | | |  | | | |  |
| --- | --- | --- | --- | --- | --- | --- | --- | --- | --- | --- | --- | --- | --- |
| Antibiotic |  |  |  |  | 19-59 y | | | | ≥ 60 y | | | |  |
|  | n | %R | %I | %S | n | %R | %I | %S | n | %R | %I | %S | p |
| *E. coli* | | | | | | | | | | | | | |
| CZT | 25 | 8.0 | 0.0 | 92.0 | 220 | 12.3 | 0.9 | 86.8 | 193 | 8.8 | 1.6 | 89.6 | ND |
| AMP | 72 | 68.1 | 0.0 | 31.9 | 400 | 73.3 | 0.8 | 26.0 | 502 | 80.1 | 1.0 | 18.9 | 0.013 |
| AMC | 102 | 19.6 | 6.9 | 73.5 | 349 | 21.8 | 11.2 | 67.0 | 317 | 25.2 | 15.8 | 59.0 | 0.396 |
| CZA | 45 | 4.4 | 0.0 | 95.6 | 347 | 7.5 | 0.0 | 92.5 | 420 | 4.8 | 0.0 | 95.2 | 0.255 |
| SAM | 407 | 41.8 | 18.7 | 39.6 | 2,561 | 39.9 | 16.6 | 43.5 | 2,607 | 41.3 | 17.2 | 41.5 | 0.544 |
| CXM | 61 | 37.7 | 8.2 | 54.1 | 298 | 39.6 | 13.4 | 47.0 | 277 | 44.4 | 10.5 | 45.1 | 0.413 |
| CAZ | 444 | 28.6 | 14.6 | 56.8 | 2,726 | 33.2 | 12.9 | 54.0 | 2,777 | 35.6 | 12.1 | 52.3 | **0.008** |
| CRO | 444 | 49.1 | 0.9 | 50.0 | 2,710 | 52.6 | 0.1 | 47.2 | 2,797 | 54.7 | 0.2 | 45.1 | 0.054 |
| FEP | 442 | 35.7 | 5.4 | 58.9 | 2,742 | 36.5 | 9.4 | 54.1 | 2,775 | 39.5 | 9.2 | 51.3 | 0.050 |
| FOX | 60 | 10.0 | 3.3 | 86.7 | 379 | 11.1 | 2.6 | 86.3 | 442 | 10.6 | 6.1 | 83.3 | 0.959 |
| ATM | 58 | 29.3 | 0.0 | 70.7 | 354 | 48.0 | 2.0 | 50.0 | 441 | 56.9 | 4.5 | 38.5 | **<0.001** |
| ETP | 439 | 2.3 | 1.1 | 96.6 | 2,749 | 2.9 | 0.5 | 96.5 | 2,802 | 3.1 | 0.9 | 96.0 | 0.660 |
| IPM | 167 | 3.6 | 0.6 | 95.8 | 1,002 | 3.0 | 0.6 | 96.4 | 1,043 | 3.5 | 0.7 | 95.9 | 0.817 |
| MEM | 448 | 1.6 | 0.4 | 98.0 | 2,767 | 2.2 | 0.2 | 97.5 | 2,821 | 2.2 | 0.2 | 97.6 | 0.652 |
| AMK | ND | ND | ND | ND | 2,816 | 6.7 | 3.8 | 80.1 | ND | ND | ND | ND | ND |
| GEN | ND | ND | ND | ND | 2,626 | 26.9 | 1.0 | 63.5 | ND | ND | ND | ND | ND |
| CIP | ND | ND | ND | ND | 2,821 | 60.8 | 16.8 | 20.1 | ND | ND | ND | ND | ND |
| LVX | ND | ND | ND | ND | 482 | 51.7 | 4.6 | 17.4 | ND | ND | ND | ND | ND |
| SXT | 372 | 60.8 | 0.0 | 39.2 | 2,289 | 56.3 | 0.0 | 43.7 | 2,408 | 53.1 | 0.0 | 46.9 | **0.007** |
| *K. pneumoniae* | | | | | | | | | | | | | |
| CZT | 13 | 7.7 | 7.7 | 84.6 | 68 | 10.3 | 1.5 | 88.2 | 56 | 12.5 | 3.6 | 83.9 | ND |
| AMC | 39 | 20.5 | 5.1 | 74.4 | 74 | 40.5 | 10.8 | 48.6 | 61 | 37.7 | 1.6 | 60.7 | 0.091 |
| CZA | 12 | 8.3 | 0.0 | 91.7 | 103 | 2.9 | 0.0 | 97.1 | 99 | 8.1 | 0.0 | 91.9 | 0.256 |
| SAM | 157 | 60.5 | 7.0 | 32.5 | 625 | 44.0 | 7.2 | 48.8 | 520 | 42.7 | 7.7 | 49.6 | **0.001** |
| CXM | ND | ND | ND | ND | 47 | 42.6 | 4.3 | 53.2 | 55 | 34.5 | 25.5 | 40.0 | ND |
| CAZ | 170 | 45.3 | 22.4 | 32.4 | 644 | 33.2 | 12.4 | 54.3 | 535 | 33.8 | 12.0 | 54.2 | **0.010** |
| CRO | 157 | 65.0 | 0.0 | 35.0 | 646 | 49.7 | 0.2 | 50.2 | 538 | 49.4 | 0.0 | 50.6 | **0.001** |
| FEP | 170 | 54.7 | 7.1 | 38.2 | 667 | 37.3 | 4.3 | 58.3 | 537 | 36.7 | 4.5 | 58.8 | **<0.001** |
| FOX | 24 | 16.7 | 8.3 | 75.0 | 111 | 11.7 | 0.0 | 88.3 | 88 | 17.0 | 5.7 | 77.3 | 0.534 |
| ATM | 28 | 67.9 | 0.0 | 32.1 | 106 | 43.4 | 0.9 | 55.7 | 101 | 53.5 | 2.0 | 44.6 | 0.053 |
| ETP | 169 | 7.7 | 1.8 | 90.5 | 664 | 6.2 | 0.8 | 93.1 | 551 | 5.1 | 1.3 | 93.6 | 0.422 |
| IPM | 96 | 6.3 | 0.0 | 93.8 | 304 | 5.6 | 1.3 | 93.1 | 241 | 6.2 | 1.2 | 92.5 | 0.943 |
| MEM | 169 | 4.7 | 0.6 | 94.7 | 675 | 5.6 | 0.4 | 93.9 | 555 | 4.3 | 0.0 | 95.7 | 0.571 |
| SXT | 110 | 56.4 | 0.0 | 43.6 | 521 | 53.4 | 0.2 | 46.4 | 455 | 46.8 | 0.0 | 53.2 | 0.060 |
| *E. cloacae* | | | | | | | | | | | | | |
| CZT | ND | ND | ND | ND | 12 | 25.0 | 0.0 | 75.0 | 10 | 30.0 | 0.0 | 70.0 | ND |
| CZA | ND | ND | ND | ND | 21 | 4.8 | 0.0 | 95.2 | 20 | 5.0 | 0.0 | 95.0 | ND |
| FEP | 57 | 12.3 | 3.5 | 84.2 | 179 | 15.6 | 8.4 | 76.0 | 118 | 26.3 | 5.1 | 68.6 | **0.029** |
| ETP | 56 | 5.4 | 3.6 | 91.1 | 178 | 15.7 | 3.9 | 80.3 | 122 | 15.6 | 6.6 | 77.9 | 0.125 |
| IPM | 28 | 0.0 | 0.0 | 100.0 | 97 | 1.0 | 3.1 | 95.9 | 57 | 8.8 | 14.0 | 77.2 | ND |
| MEM | 56 | 5.4 | 0.0 | 94.6 | 179 | 6.1 | 0.6 | 93.3 | 123 | 5.7 | 0.8 | 93.5 | 0.971 |
| SXT | 35 | 22.9 | 0.0 | 77.1 | 120 | 20.8 | 0.0 | 79.2 | 95 | 30.5 | 1.1 | 68.4 | 0.252 |
| *A. baumannii* | | | | | | | | | | | | | |
| SAM | 50 | 28.0 | 10.0 | 62.0 | 224 | 54.0 | 17.4 | 28.6 | 99 | 51.5 | 21.2 | 27.3 | **0.00**4 |
| TZP | 34 | 35.3 | 0.0 | 64.7 | 112 | 81.3 | 0.0 | 18.8 | 55 | 78.2 | 0.0 | 21.8 | **0.001** |
| FEP | 51 | 21.6 | 17.6 | 60.8 | 226 | 48.2 | 26.1 | 25.7 | 102 | 48.0 | 27.5 | 24.5 | **0.002** |
| IPM | 40 | 30.0 | 0.0 | 70.0 | 154 | 76.6 | 0.0 | 23.4 | 76 | 76.3 | 0.0 | 23.7 | **<0.001** |
| MEM | 52 | 34.6 | 0.0 | 65.4 | 229 | 72.5 | 0.0 | 27.5 | 103 | 72.8 | 1.0 | 26.2 | **<0.001** |
| AMK | 27 | 14.8 | 0.0 | 85.2 | 141 | 51.1 | 9.2 | 39.7 | 74 | 60.8 | 9.5 | 29.7 | **<0.001** |
| GEN | 43 | 27.9 | 9.3 | 62.8 | 208 | 53.4 | 11.1 | 35.6 | 100 | 59.0 | 14.0 | 27.0 | **0.002** |
| TOB | ND | ND | ND | ND | 43 | 79.1 | 0.0 | 20.9 | 21 | 76.2 | 0.0 | 23.8 | ND |
| CIP | 52 | 36.5 | 0.0 | 63.5 | 230 | 73.9 | 1.3 | 24.8 | 103 | 76.7 | 1.9 | 21.4 | **<0.001** |
| LVX | ND | ND | ND | ND | 42 | 73.8 | 2.4 | 23.8 | 26 | 76.9 | 0.0 | 23.1 | 0.773 |
| SXT | 18 | 38.9 | 0.0 | 61.1 | 111 | 65.8 | 0.9 | 33.3 | 50 | 74.0 | 0.0 | 26.0 | **0.027** |
| *P. aeruginosa* | | | | | | | | | | | | | |
| CZT | 50 | 2.0 | 2.0 | 96.0 | 184 | 13.6 | 3.3 | 83.2 | 114 | 13.2 | 1.8 | 85.1 | 0.068 |
| CZA | 54 | 11.1 | 0.0 | 88.9 | 219 | 19.2 | 0.0 | 80.8 | 177 | 26.0 | 0.0 | 74.0 | **0.043** |
| TZP | 118 | 24.6 | 1.7 | 73.7 | 408 | 27.5 | 8.3 | 64.2 | 355 | 30.4 | 5.4 | 64.2 | 0.419 |
| CAZ | 168 | 17.9 | 5.4 | 76.8 | 613 | 27.6 | 4.7 | 67.7 | 502 | 28.1 | 2.4 | 69.5 | **0.024** |
| FEP | 166 | 12.7 | 7.2 | 80.1 | 610 | 18.5 | 10.7 | 70.8 | 500 | 20.2 | 9.6 | 70.2 | 0.094 |
| ATM | 29 | 34.5 | 10.3 | 55.2 | 121 | 42.1 | 9.9 | 47.9 | 118 | 36.4 | 8.5 | 55.1 | 0.584 |
| IPM | 130 | 40.8 | 4.6 | 54.6 | 427 | 36.5 | 4.0 | 59.5 | 369 | 29.0 | 5.7 | 65.3 | **0.019** |
| MEM | 168 | 28.0 | 7.1 | 64.9 | 612 | 32.4 | 6.7 | 60.9 | 501 | 27.3 | 4.6 | 68.1 | 0.164 |
| AMK | 67 | 19.4 | 6.0 | 74.6 | 263 | 19.0 | 2.7 | 78.3 | 282 | 17.4 | 3.9 | 78.7 | 0.859 |

CZT: Ceftolozane/Tazobactam, AMP: Ampicillin, AMC: Amoxicillin/Clavulanic acid, CZA: Ceftazidime/Avibactam, SAM: Ampicillin/Sulbactam, CXM: Cefuroxime, CAZ: Ceftazidime, CRO: Ceftriaxone, FEP: Cefepime, FOX: Cefoxitin, ATM: Aztreonam, ETP: Ertapenem, IPM: Imipenem, MEM: Meropenem, AMK: Amikacin, GEN: Gentamicin, CIP: Ciprofloxacin, LVX: Levofloxacin, SXT: Sulfamethoxazole/Trimethoprim, TZP: Piperacillin/Tazobactam, TOB: Tobramycin. ND: not determined.
