# Supplementary material for: The threat of multidrug-resistant microorganisms: active surveillance of key antimicrobial resistant pathogens in 2025 - a report from the INVIFAR network
Source: Eur J Clin Microbiol Infect Dis. 2026 Jan 6;45(4):1041–57. doi: 10.1007/s10096-025-05330-2 (PMC13086762; doi:10.1007/s10096-025-05330-2)
Supplement: Supplementary file 3 — Supplementary Material 3 [file 10096_2025_5330_MOESM3_ESM.docx]

Suppl Table 3. Distribution of antibiotic resistance according to site of attention

| **Antibiotic** | **EME** | | | | **INX** | | | | **ICU** | | | | **OUT** | | | | **p** |
| --- | --- | --- | --- | --- | --- | --- | --- | --- | --- | --- | --- | --- | --- | --- | --- | --- | --- |
|  | **n** | **%R** | **%I** | **%S** | **n** | **%R** | **%I** | **%S** | **n** | **%R** | **%I** | **%S** | **n** | **%R** | **%I** | **%S** |  |
| *E. coli* | | | | | | | | | | | | | | | | | |
| CZT | 71 | 12.7 | 2.8 | 84.5 | 287 | 11.1 | 0.7 | 88.2 | 30 | 10.0 | 3.3 | 86.7 | 60 | 5.0 | 0.0 | 95.0 | 0.488 |
| AMP | 177 | 75.1 | 0.6 | 24.3 | 333 | 82.0 | 0.6 | 17.4 | 54 | 77.8 | 0.0 | 22.2 | 429 | 73.4 | 1.2 | 25.4 | 0.045 |
| AMC | 55 | 9.1 | 10.9 | 80.0 | 230 | 28.3 | 9.1 | 62.6 | 16 | 43.8 | 6.3 | 50.0 | 477 | 22.4 | 14.3 | 63.3 | ND |
| CZA | 167 | 4.8 | 0.0 | 95.2 | 405 | 7.4 | 0.0 | 92.6 | 38 | 7.9 | 0.0 | 92.1 | 214 | 3.3 | 0.0 | 96.7 | 0.171 |
| SAM | 849 | 42.9 | 20.5 | 36.6 | 2,850 | 44.7 | 15.1 | 40.2 | 166 | 48.2 | 17.5 | 34.3 | 2,107 | 32.2 | 17.7 | 50.1 | **<0.001** |
| CXM | 57 | 42.1 | 3.5 | 54.4 | 179 | 35.2 | 2.8 | 62.0 | 20 | 15.0 | 5.0 | 80.0 | 383 | 48.6 | 15.9 | 35.5 | ND |
| CAZ | 895 | 38.9 | 15.0 | 46.1 | 2,967 | 39.2 | 14.0 | 46.8 | 172 | 46.5 | 11.6 | 41.9 | 2,318 | 22.6 | 9.6 | 67.8 | **<0.001** |
| CRO | 840 | 60.6 | 0.7 | 38.7 | 2,846 | 60.9 | 0.1 | 39.0 | 173 | 68.8 | 0.0 | 31.2 | 2,485 | 39.7 | 0.2 | 60.1 | **<0.001** |
| FEP | 888 | 43.1 | 8.8 | 48.1 | 3,012 | 44.0 | 9.3 | 46.6 | 185 | 52.4 | 9.7 | 37.8 | 2,279 | 25.5 | 7.8 | 66.7 | **<0.001** |
| FOX | 151 | 11.3 | 8.6 | 80.1 | 319 | 13.2 | 3.1 | 83.7 | 46 | 15.2 | 4.3 | 80.4 | 381 | 8.1 | 3.7 | 88.2 | 0.132 |
| ATM | 172 | 57.0 | 3.5 | 39.5 | 385 | 53.5 | 2.1 | 44.4 | 40 | 70.0 | 2.5 | 27.5 | 269 | 43.5 | 4.5 | 52.0 | **0.002** |
| ETP | 893 | 3.0 | 0.8 | 96.2 | 2,978 | 4.0 | 0.8 | 95.2 | 185 | 5.9 | 1.6 | 92.4 | 2,336 | 1.3 | 0.5 | 98.2 | **<0.001** |
| IPM | 386 | 2.8 | 0.3 | 96.9 | 1,132 | 4.2 | 0.7 | 95.1 | 123 | 7.3 | 0.0 | 92.7 | 605 | 1.3 | 0.8 | 97.9 | **<0.001** |
| MEM | 899 | 1.9 | 0.7 | 97.4 | 2,996 | 3.0 | 0.2 | 96.8 | 185 | 4.9 | 1.1 | 94.1 | 2,366 | 1.0 | 0.0 | 99.0 | **<0.001** |
| SXT | 693 | 57.4 | 0.0 | 42.6 | 2,296 | 58.1 | 0.0 | 41.9 | 131 | 58.8 | 0.0 | 41.2 | 2,343 | 50.2 | 0.0 | 49.7 | **<0.001** |
| *K. pneumoniae* | | | | | | | | | | | | | | | | | |
| CZT | 18 | 5.6 | 0.0 | 94.4 | 93 | 15.1 | 4.3 | 80.6 | 19 | 0.0 | 0.0 | 100.0 | ND | ND | ND | ND | 0.120 |
| AMC | 11 | 0.0 | 0.0 | 100.0 | 80 | 40.0 | 6.3 | 53.8 | ND | ND | ND | ND | 73 | 34.2 | 8.2 | 57.5 | **0.033** |
| CZA | 30 | 3.3 | 0.0 | 96.7 | 126 | 7.9 | 0.0 | 92.1 | 26 | 0.0 | 0.0 | 100.0 | 36 | 2.8 | 0.0 | 97.2 | 0.292 |
| SAM | 176 | 41.5 | 10.2 | 48.3 | 711 | 51.6 | 7.6 | 40.8 | 156 | 46.8 | 5.8 | 47.4 | 300 | 31.0 | 5.0 | 64.0 | **<0.001** |
| CXM | ND | ND | ND | ND | 39 | 38.5 | 10.3 | 51.3 | 14 | 7.1 | 0.0 | 92.9 | 58 | 46.6 | 22.4 | 31.0 | ND |
| CAZ | 179 | 31.3 | 10.1 | 58.7 | 735 | 40.7 | 14.8 | 44.5 | 164 | 36.0 | 20.1 | 43.9 | 326 | 22.4 | 8.3 | 69.3 | **<0.001** |
| CRO | 162 | 45.1 | 0.0 | 54.9 | 711 | 59.9 | 0.0 | 40.1 | 157 | 54.8 | 0.6 | 44.6 | 360 | 35.3 | 0.0 | 64.7 | **<0.001** |
| FEP | 174 | 31.0 | 4.0 | 64.9 | 761 | 46.6 | 4.9 | 48.5 | 168 | 41.1 | 7.1 | 51.8 | 323 | 23.8 | 3.4 | 72.8 | **<0.001** |
| FOX | 22 | 13.6 | 4.5 | 81.8 | 118 | 13.6 | 2.5 | 83.9 | 25 | 20.0 | 12.0 | 68.0 | 61 | 13.1 | 0.0 | 86.9 | ND |
| ATM | 33 | 45.5 | 0.0 | 54.5 | 134 | 56.7 | 2.2 | 41.0 | 31 | 64.5 | 0.0 | 35.5 | 42 | 26.2 | 0.0 | 73.8 | **0.002** |
| ETP | 177 | 4.5 | 1.1 | 94.4 | 755 | 7.4 | 1.2 | 91.4 | 168 | 7.1 | 0.0 | 92.9 | 337 | 2.1 | 1.5 | 96.4 | **0.004** |
| IPM | 76 | 2.6 | 1.3 | 96.1 | 369 | 8.4 | 1.4 | 90.2 | 100 | 4.0 | 2.0 | 94.0 | 108 | 1.9 | 0.0 | 98.1 | **0.025** |
| MEM | 179 | 2.2 | 0.0 | 97.8 | 762 | 6.0 | 0.4 | 93.6 | 167 | 5.4 | 0.6 | 94.0 | 345 | 2.0 | 0.0 | 98.0 | **0.010** |
| SXT | 132 | 44.7 | 0.0 | 55.3 | 546 | 57.5 | 0.2 | 42.3 | 113 | 51.3 | 0.0 | 48.7 | 339 | 41.0 | 0.0 | 59.0 | **<0.001** |
| *E. cloacae* | | | | | | | | | | | | | | | | | |
| CZT | ND | ND | ND | ND | 17 | 29.4 | 0.0 | 70.6 | ND | ND | ND | ND | ND | ND | ND | ND | ND |
| CZA | ND | ND | ND | ND | 24 | 4.2 | 0.0 | 95.8 | ND | ND | ND | ND | ND | ND | ND | ND | ND |
| FEP | 34 | 23.5 | 11.8 | 64.7 | 217 | 17.5 | 6.5 | 76.0 | 48 | 12.5 | 4.2 | 83.3 | 62 | 21.0 | 3.2 | 75.8 | **0.002** |
| ETP | 33 | 21.2 | 12.1 | 66.7 | 217 | 14.7 | 5.1 | 80.2 | 51 | 9.8 | 0.0 | 90.2 | 62 | 6.5 | 3.2 | 90.3 | 0.153 |
| IPM | 21 | 0.0 | 23.8 | 76.2 | 109 | 1.8 | 3.7 | 94.5 | 29 | 13.8 | 0.0 | 86.2 | 24 | 0.0 | 8.3 | 91.7 | ND |
| MEM | 34 | 5.9 | 0.0 | 94.1 | 217 | 5.5 | 0.9 | 93.5 | 51 | 3.9 | 0.0 | 96.1 | 63 | 4.8 | 0.0 | 95.2 | 0.965 |
| SXT | 22 | 31.8 | 0.0 | 68.2 | 141 | 29.1 | 0.7 | 70.2 | 37 | 10.8 | 0.0 | 89.2 | 57 | 19.3 | 0.0 | 80.7 | ND |
| 1. *baumannii* | | | | | | | | | | | | | | | | | |
| TZP | 28 | 57.1 | 0.0 | 42.9 | 139 | 78.4 | 0.0 | 21.6 | 48 | 77.1 | 0.0 | 22.9 | ND | ND | ND | ND | ND |
| SAM | 40 | 52.5 | 7.5 | 40.0 | 235 | 49.8 | 20.4 | 29.8 | 95 | 52.6 | 18.9 | 28.4 | 24 | 20.8 | 29.2 | 50.0 | **0.039** |
| FEP | 40 | 40.0 | 22.5 | 37.5 | 238 | 45.4 | 30.3 | 24.4 | ND | ND | ND | ND | 27 | 25.9 | 18.5 | 55.6 | 0.142 |
| IPM | 35 | 51.4 | 0.0 | 48.6 | 169 | 74.6 | 0.0 | 25.4 | 67 | **79.1** | 0.0 | 20.9 | 19 | 47.4 | 0.0 | 52.6 | **0.002** |
| MEM | 40 | 57.5 | 0.0 | 42.5 | 241 | 71.4 | 0.8 | 27.8 | 95 | 69.5 | 3.2 | 27.4 | 29 | 44.8 | 0.0 | 55.2 | **0.015** |
| SXT | 11 | 81.8 | 0.0 | 18.2 | 108 | 69.4 | 0.0 | 30.6 | 57 | 68.4 | 0.0 | 31.6 | 24 | 54.2 | 4.2 | 41.7 | 0.364 |
| AMK | 26 | 42.3 | 3.8 | 53.8 | 148 | 52.7 | 12.8 | 34.5 | 72 | 41.7 | 13.9 | 44.4 | 18 | 44.4 | 5.6 | 50.0 | ND |
| GEN | 37 | 51.4 | 10.8 | 37.8 | 214 | 55.1 | 12.6 | 32.2 | 93 | 46.2 | 20.4 | 33.3 | 29 | 41.4 | 3.4 | 55.2 | 0.340 |
| CIP | 40 | 62.5 | 0.0 | 37.5 | 241 | 75.1 | 0.8 | 24.1 | 96 | 68.8 | 0.0 | 31.3 | 29 | 51.7 | 10.3 | 37.9 | **0.033** |
| LVX | ND | ND | ND | ND | 33 | 78.8 | 0.0 | 21.2 | 20 | 75.0 | 5.0 | 20.0 | 14 | 50.0 | 0.0 | 50.0 | ND |
| TOB | ND | ND | ND | ND | 32 | 84.4 | 0.0 | 15.6 | 18 | 83.3 | 0.0 | 16.7 | ND | ND | ND | ND | ND |
| *P. aeruginosa* | | | | | | | | | | | | | | | | | |
| CZT | 35 | 5.7 | 0.0 | 94.3 | 213 | 13.1 | 3.8 | 83.1 | 55 | 9.1 | 0.0 | 90.9 | 52 | 13.5 | 1.9 | 84.6 | 0.545 |
| CZA | 72 | 13.9 | 0.0 | 86.1 | 261 | 24.9 | 0.0 | 75.1 | 64 | 20.3 | 0.0 | 79.7 | 61 | 11.5 | 0.0 | 88.5 | 0.046 |
| TZP | 121 | 25.6 | 7.4 | 66.9 | 576 | 30.2 | 6.3 | 63.5 | 104 | 29.8 | 2.9 | 67.3 | 118 | 19.5 | 8.5 | 72.0 | 0.107 |
| CAZ | 153 | 22.2 | 1.3 | 76.5 | 820 | 27.0 | 5.1 | 67.9 | 144 | 27.1 | 2.8 | 70.1 | 196 | 26.0 | 2.6 | 71.4 | 0.672 |
| FEP | 154 | 14.9 | 7.8 | 77.3 | ND | ND | ND | ND | ND | ND | ND | ND | 195 | 17.4 | 6.2 | 76.4 | 0.530 |
| ATM | 44 | 34.1 | 6.8 | 59.1 | 179 | 39.7 | 8.9 | 51.4 | 21 | 38.1 | 9.5 | 52.4 | 26 | 42.3 | 15.4 | 42.3 | ND |
| IPM | 122 | 26.2 | 7.4 | 66.4 | 602 | 34.9 | 3.7 | 61.5 | 113 | 32.7 | 3.5 | 63.7 | 126 | 35.7 | 7.9 | 56.3 | 0.296 |
| MEM | 153 | 19.6 | 5.9 | 74.5 | 819 | 30.5 | 5.5 | 64.0 | 144 | 32.6 | 2.8 | 64.6 | 195 | 29.2 | 8.7 | 62.1 | **0.040** |
| AMK | 75 | 10.7 | 2.7 | 86.7 | 355 | 18.0 | 2.3 | 79.7 | 62 | 12.9 | 4.8 | 82.3 | 137 | 20.4 | 7.3 | 72.3 | 0.237 |

Ceftolozane/Tazobactam, AMP: Ampicillin, AMC: Amoxicillin/Clavulanic acid, CZA: Ceftazidime/Avibactam, SAM: Ampicillin/Sulbactam, CXM: Cefuroxime, CAZ: Ceftazidime, CRO: Ceftriaxone, FEP: Cefepime, FOX: Cefoxitin, ATM: Aztreonam, ETP: Ertapenem, IPM: Imipenem, MEM: Meropenem, AMK: Amikacin, GEN: Gentamicin, CIP: Ciprofloxacin, LVX: Levofloxacin, SXT: Ssulfamethoxazole/Trimethoprim, TZP: Piperacillin/Tazobactam, TOB: Tobramycin. EME: emergency department, INX: non-ICU inpatient wards, ICU: intensive care unit, and OUT: outpatient settings. ND: Not Determined.
