# Supplementary material for: The threat of multidrug-resistant microorganisms: active surveillance of key antimicrobial resistant pathogens in 2025 - a report from the INVIFAR network
Source: Eur J Clin Microbiol Infect Dis. 2026 Jan 6;45(4):1041–57. doi: 10.1007/s10096-025-05330-2 (PMC13086762; doi:10.1007/s10096-025-05330-2)
Supplement: Supplementary file 4 — Supplementary Material 4 [file 10096_2025_5330_MOESM4_ESM.docx]

Suppl Table 4. Distribution of drug resistance according to clinical specimen.

|  | **Urine** | | | | **Respiratory** | | | | **Blood** | | | |  |
| --- | --- | --- | --- | --- | --- | --- | --- | --- | --- | --- | --- | --- | --- |
| **Antibiotic** | **n** | **%R** | **%I** | **%S** | **n** | **%R** | **%I** | **%S** | **n** | **%R** | **%I** | **%S** | **p** |
| *E. coli* | | | | | | | | | | | | | |
| CZT | 173 | 6.9 | 0.6 | 92.5 | 16 | 18.8 | 6.3 | 75.0 | 171 | 11.1 | 1.2 | 87.7 | ND |
| AMP | 739 | 73.2 | 0.9 | 25.8 | 34 | 82.4 | 0.0 | 17.6 | 56 | 85.7 | 1.8 | 12.5 | 0.066 |
| AMC | 617 | 20.3 | 13.3 | 66.5 | 33 | 42.4 | 3.0 | 54.5 | 21 | 19.0 | 14.3 | 66.7 | ND |
| CZA | 462 | 6.7 | 0.0 | 93.3 | 26 | 0.0 | 0.0 | 100.0 | 186 | 3.8 | 0.0 | 96.2 | 0.151 |
| SAM | 4,524 | 37.9 | 17.1 | 45.0 | 143 | 50.3 | 19.6 | 30.1 | 428 | 46.7 | 16.8 | 36.4 | **<0.001** |
| CXM | 529 | 40.8 | 11.5 | 47.6 | ND | ND | ND | ND | 19 | 21.1 | 0.0 | 78.9 | 0.084 |
| CAZ | 4,844 | 28.9 | 11.6 | 59.5 | 147 | 57.8 | 13.6 | 28.6 | 436 | 40.6 | 16.5 | 42.9 | **<0.001** |
| CRO | 4,983 | 47.2 | 0.3 | 52.6 | 154 | 81.8 | 0.0 | 18.2 | 288 | 71.2 | 0.0 | 28.8 | **<0.001** |
| FEP | 4,801 | 32.4 | 8.2 | 59.4 | 170 | 64.7 | 10.6 | 24.7 | 443 | 46.5 | 9.0 | 44.5 | **<0.001** |
| FOX | 643 | 7.8 | 5.0 | 87.2 | 46 | 19.6 | 2.2 | 78.3 | 47 | 14.9 | 4.3 | 80.9 | **0.008** |
| ATM | 489 | 48.1 | 4.1 | 47.9 | 35 | 71.4 | 8.6 | 20.0 | 186 | 43.0 | 0.0 | 57.0 | **0.008** |
| ETP | 4,829 | 1.8 | 0.7 | 97.5 | 170 | 8.8 | 1.2 | 90.0 | 444 | 5.6 | 0.5 | 93.9 | **<0.001** |
| IPM | 1,205 | 2.1 | 0.7 | 97.3 | 121 | 5.8 | 0.8 | 93.4 | 352 | 4.0 | 0.0 | 96.0 | **0.016** |
| MEM | 4,874 | 1.4 | 0.1 | 98.5 | 170 | 5.3 | 0.0 | 94.7 | 445 | 3.6 | 0.2 | 96.2 | **<0.001** |
| SXT | 4,599 | 53.1 | 0.0 | 46.9 | 105 | 67.6 | 0.0 | 32.4 | 164 | 62.8 | 0.0 | 37.2 | **<0.001** |
| NOR | 3,128 | 53.3 | 2.7 | 44.0 | ND | ND | ND | ND | ND | ND | ND | ND | ND |
| NIT | 4,556 | 6.2 | 5.4 | 88.4 | ND | ND | ND | ND | ND | ND | ND | ND | ND |
| *K. pneumoniae* | | | | | | | | | | | | | |
| CZT | 43 | 9.3 | 0.0 | 90.7 | 27 | 11.1 | 7.4 | 81.5 | 36 | 8.3 | 0.0 | 91.7 | ND |
| AMC | 99 | 27.3 | 7.1 | 65.7 | 33 | 33.3 | 6.1 | 60.6 | 12 | 66.7 | 0.0 | 33.3 | ND |
| CZA | 90 | 5.6 | 0.0 | 94.4 | 30 | 6.7 | 0.0 | 93.3 | 46 | 0.0 | 0.0 | 100.0 | 0.238 |
| SAM | 700 | 41.4 | 7.1 | 51.4 | 177 | 43.5 | 5.6 | 50.8 | 183 | 52.5 | 4.9 | 42.6 | **0.028** |
| CAZ | 738 | 31.6 | 10.3 | 58.1 | 188 | 39.4 | 11.7 | 48.9 | 189 | 32.8 | 23.3 | 43.9 | 0.128 |
| CRO | 758 | 44.9 | 0.0 | 55.1 | 191 | 51.8 | 0.5 | 47.6 | 160 | 65.0 | 0.0 | 35.0 | **<0.001** |
| FEP | 728 | 34.2 | 3.8 | 62.0 | 204 | 43.1 | 3.4 | 53.4 | 191 | 45.5 | 6.8 | 47.6 | **0.003** |
| FOX | 96 | 14.6 | 2.1 | 83.3 | 50 | 16.0 | 6.0 | 78.0 | 26 | 11.5 | 7.7 | 80.8 | ND |
| ATM | 91 | 47.3 | 1.1 | 51.6 | 36 | 66.7 | 0.0 | 33.3 | 52 | 38.5 | 3.8 | 57.7 | **0.034** |
| ETP | 738 | 4.3 | 1.9 | 93.8 | 204 | 10.8 | 0.0 | 89.2 | 193 | 4.7 | 0.0 | 95.3 | **0.001** |
| IPM | 183 | 4.9 | 2.2 | 92.9 | 140 | 8.6 | 1.4 | 90.0 | 142 | 2.8 | 0.0 | 97.2 | 0.096 |
| MEM | 751 | 3.1 | 0.1 | 96.8 | 203 | 9.9 | 0.5 | 89.7 | 192 | 4.2 | 1.0 | 94.8 | **<0.001** |
| SXT | 723 | 46.3 | 0.1 | 53.5 | 132 | 52.3 | 0.0 | 47.7 | 87 | 62.1 | 0.0 | 37.9 | **0.014** |
| NOR | 431 | 21.3 | 4.6 | 74.0 | ND | ND | ND | ND | ND | ND | ND | ND | ND |
| NIT | 699 | 31.0 | 44.5 | 24.5 | ND | ND | ND | ND | ND | ND | ND | ND | ND |
| *E. cloacae* | | | | | | | | | | | | | |
| CZA | ND | ND | ND | ND | ND | ND | ND | ND | 11 | 0.0 | 0.0 | 100.0 | ND |
| TZP | ND | ND | ND | ND | ND | ND | ND | ND | 49 | 22.4 | 6.1 | 71.4 | ND |
| CAZ | 113 | 46.9 | 1.8 | 51.3 | 48 | 22.9 | 2.1 | 75.0 | 83 | 30.1 | 1.2 | 68.7 | **0.005** |
| CRO | 108 | 51.9 | 1.9 | 46.3 | 49 | 32.7 | 0.0 | 67.3 | 73 | 35.6 | 0.0 | 64.4 | **0.027** |
| FEP | 114 | 33.3 | 6.1 | 60.5 | 58 | 10.3 | 5.2 | 84.5 |  |  |  |  | **0.001** |
| ETP | 114 | 21.1 | 7.9 | 71.1 | 58 | 8.6 | 5.2 | 86.2 | 84 | 10.7 | 3.6 | 85.7 | **0.041** |
| IPM | 21 | 0.0 | 23.8 | 76.2 | 46 | 2.2 | 2.2 | 95.7 | 52 | 5.8 | 3.8 | 90.4 | ND |
| MEM | 116 | 11.2 | 1.7 | 87.1 | 58 | 5.2 | 0.0 | 94.8 | 84 | 2.4 | 0.0 | 97.6 | **0.044** |
| SXT | 109 | 34.9 | 0.9 | 64.2 | 36 | 13.9 | 0.0 | 86.1 | 39 | 15.4 | 0.0 | 84.6 | **0.010** |
| NOR | 86 | 31.4 | 3.5 | 65.1 | ND | ND | ND | ND | ND | ND | ND | ND | ND |
| NIT | 104 | 38.5 | 34.6 | 26.9 | ND | ND | ND | ND | ND | ND | ND | ND | ND |
| *A. baumannii* | | | | | | | | | | | | | |
| SAM | 29 | 31.0 | 17.2 | 51.7 | 173 | 55.5 | 20.8 | 23.7 | 85 | 40.0 | 12.9 | 47.1 | **0.009** |
| TZP | ND | ND | ND | ND | 118 | 77.1 | 0.8 | 22.0 | 48 | 60.4 | 0.0 | 39.6 | **0.029** |
| CAZ | 29 | 65.5 | 3.4 | 31.0 | 176 | 76.1 | 1.7 | 22.2 | 86 | 54.7 | 4.7 | 40.7 | **0.002** |
| FEP | 29 | 37.9 | 20.7 | 41.4 | 175 | 49.7 | 26.3 | 24.0 | 84 | 35.7 | 17.9 | 46.4 | ND |
| IPM | 10 | 40.0 | 0.0 | 60.0 | 145 | 75.9 | 0.0 | 24.1 | 64 | 53.1 | 0.0 | 46.9 | ND |
| MEM | 31 | 51.6 | 0.0 | 48.4 | 174 | 73.6 | 1.7 | 24.7 | 87 | 51.7 | 1.1 | 47.1 | **<0.001** |
| SXT | 24 | 70.8 | 0.0 | 29.2 | 73 | 72.6 | 0.0 | 27.4 | 36 | 50.0 | 2.8 | 47.2 | ND |
| AMK | 15 | 46.7 | 6.7 | 46.7 | 117 | 55.6 | 12.0 | 32.5 | 52 | 38.5 | 5.8 | 55.8 | ND |
| GEN | 31 | 38.7 | 16.1 | 45.2 | 162 | 58.6 | 11.7 | 29.6 | 82 | 40.2 | 11.0 | 48.8 | **0.009** |
| CIP | 31 | 61.3 | 12.9 | 25.8 | 175 | 76.0 | 0.6 | 23.4 | 87 | 54.0 | 0.0 | 46.0 | **0.001** |
| TOB | ND | ND | ND | ND | 30 | 86.7 | 0.0 | 13.3 | ND | ND | ND | ND | ND |
| LVX | ND | ND | ND | ND | 37 | 73.0 | 2.7 | 24.3 | ND | ND | ND | ND | ND |
| *P. aeruginosa* | | | | | | | | | | | | | |
| CZT | 73 | 24.7 | 4.1 | 71.2 | 128 | 8.6 | 2.3 | 89.1 | 56 | 17.9 | 0.0 | 82.1 | **0.008** |
| CZA | 112 | 36.6 | 0.0 | 63.4 | 135 | 14.1 | 0.0 | 85.9 | 65 | 21.5 | 0.0 | 78.5 | **<0.001** |
| TZP | 210 | 29.5 | 7.1 | 63.3 | ND | ND | ND | ND | 126 | 26.2 | 3.2 | 70.6 | 0.511 |
| CAZ | 366 | 32.8 | 3.6 | 63.7 | 385 | 22.3 | 4.2 | 73.5 | 160 | 23.1 | 4.4 | 72.5 | **0.003** |
| FEP | 366 | 27.0 | 6.6 | 66.4 | 381 | 12.9 | 11.0 | 76.1 | 158 | 12.7 | 10.8 | 76.6 | **<0.001** |
| ATM | 55 | 63.6 | 5.5 | 30.9 | 86 | 31.4 | 7.0 | 61.6 | 31 | 29.0 | 6.5 | 64.5 | **<0.001** |
| IPM | 214 | 37.9 | 5.1 | 57.0 | 331 | 34.4 | 3.0 | 62.5 | 134 | 31.3 | 2.2 | 66.4 | 0.450 |
| MEM | 365 | 34.5 | 6.0 | 59.5 | 385 | 28.8 | 6.0 | 65.2 | 159 | 21.4 | 6.9 | 71.7 | **0.009** |
| AMK | 295 | 26.1 | 4.1 | 69.8 | 134 | 11.2 | 3.7 | 85.1 | 47 | 2.1 | 4.3 | 93.6 | **<0.001** |
| NOR | 143 | 37.8 | 3.5 | 58.7 | ND | ND | ND | ND | ND | ND | ND | ND | ND |
| NIT | 51 | 100.0 | 0.0 | 0.0 | ND | ND | ND | ND | ND | ND | ND | ND | ND |

CZT: Ceftolozane/Tazobactam, AMP: Ampicillin, AMC: Amoxicillin/Clavulanic acid, CZA: Ceftazidime/Avibactam, SAM: Ampicillin/Sulbactam, CXM: Cefuroxime, CAZ: Ceftazidime, CRO: Ceftriaxone, FEP: Cefepime, FOX: Cefoxitin, ATM: Aztreonam, ETP: Ertapenem, IPM: Imipenem, MEM: Meropenem, AMK: Amikacin, GEN: Gentamicin, CIP: Ciprofloxacin, LVX: Levofloxacin, SXT: Sulfamethoxazole/Trimethoprim, TZP: Piperacillin/Tazobactam, TOB: Tobramycin, NOR: Norfloxacin, NIT: Nitrofurantoin. ND: Not Determined.
