# Supplementary material for: The threat of multidrug-resistant microorganisms: active surveillance of key antimicrobial resistant pathogens in 2025 - a report from the INVIFAR network
Source: Eur J Clin Microbiol Infect Dis. 2026 Jan 6;45(4):1041–57. doi: 10.1007/s10096-025-05330-2 (PMC13086762; doi:10.1007/s10096-025-05330-2)
Supplement: Supplementary file 5 — Supplementary Material 5 [file 10096_2025_5330_MOESM5_ESM.docx]

Suppl Table 5. Distribution of antibiotic resistance in clinical isolates recovered from biopsies or abscess.

|  | **Biopsies** | | | | **Abscess** | | | |
| --- | --- | --- | --- | --- | --- | --- | --- | --- |
| **Antibiotic** | **n** | **%R** | **%I** | **%S** | **n** | **%R** | **%I** | **%S** |
| *E. coli* | | | | | | | | |
| CZT | 38 | 13.2 | 0.0 | 86.8 | 33 | 12.1 | 0.0 | 87.9 |
| AMP | ND | ND | ND | ND | 44 | 86.4 | 0.0 | 13.6 |
| CZA | 33 | 9.1 | 0.0 | 90.9 | 53 | 3.8 | 0.0 | 96.2 |
| SAM | 208 | 43.8 | 14.9 | 41.3 | 182 | 47.3 | 15.9 | 36.8 |
| TZP | 185 | 20.0 | 1.6 | 78.4 | ND | ND | ND | ND |
| CAZ | 213 | 48.4 | 9.9 | 41.8 | 187 | 46.5 | 15.5 | 38.0 |
| CRO | 213 | 64.3 | 0.0 | 35.7 | 170 | 71.2 | 0.0 | 28.8 |
| FEP | 231 | 51.5 | 8.2 | 40.3 | 189 | 53.4 | 10.6 | 36.0 |
| FOX | 33 | 21.2 | 0.0 | 78.8 | 43 | 23.3 | 7.0 | 69.8 |
| ATM | 35 | 65.7 | 2.9 | 31.4 | 56 | 64.3 | 3.6 | 32.1 |
| ETP | 232 | 5.2 | 0.4 | 94.4 | 190 | 2.6 | 1.1 | 96.3 |
| IPM | 191 | 4.2 | 0.0 | 95.8 | 158 | 2.5 | 1.9 | 95.6 |
| MEM | 233 | 4.7 | 0.0 | 95.3 | 190 | 2.6 | 0.0 | 97.4 |
| AMK | 213 | 5.2 | 1.4 | 93.4 | ND | ND | ND | ND |
| GEN | 189 | 32.8 | 2.6 | 64.6 | ND | ND | ND | ND |
| CIP | 231 | 66.7 | 17.3 | 16.0 | ND | ND | ND | ND |
| SXT | 80 | 63.8 | 0.0 | 36.3 | 85 | 58.8 | 0.0 | 41.2 |
| *K. pneumoniae* | | | | | | | | |
| AMC | 14 | 64.3 | 0.0 | 35.7 | 11 | 81.8 | 0.0 | 18.2 |
| SAM | 63 | 50.8 | 7.9 | 41.3 | 74 | 41.9 | 9.5 | 48.6 |
| TZP | 68 | 20.6 | 16.2 | 63.2 | ND | ND | ND | ND |
| CAZ | 68 | 36.8 | 14.7 | 48.5 | 75 | 34.7 | 10.7 | 54.7 |
| CRO | 78 | 59.0 | 0.0 | 41.0 | 67 | 50.7 | 0.0 | 49.3 |
| FEP | 82 | 41.5 | 2.4 | 56.1 | 76 | 34.2 | 6.6 | 59.2 |
| FOX | 21 | 9.5 | 0.0 | 90.5 | 11 | 18.2 | 0.0 | 81.8 |
| ETP | 81 | 3.7 | 0.0 | 96.3 | 77 | 6.5 | 0.0 | 93.5 |
| IPM | 69 | 2.9 | 0.0 | 97.1 | 47 | 2.1 | 2.1 | 95.7 |
| MEM | 82 | 2.4 | 0.0 | 97.6 | 77 | 3.9 | 0.0 | 96.1 |
| GEN | 63 | 39.7 | 3.2 | 57.1 | ND | ND | ND | ND |
| CIP | 82 | 52.4 | 9.8 | 37.8 | ND | ND | ND | ND |
| SXT | 38 | 68.4 | 0.0 | 31.6 | 45 | 42.2 | 0.0 | 57.8 |
| *E. cloacae* | | | | | | | | |
| TZP | 30 | 23.3 | 6.7 | 70.0 | ND | ND | ND | ND |
| FEP | 35 | 11.4 | 2.9 | 85.7 | ND | ND | ND | ND |
| ETP | 35 | 8.6 | 2.9 | 88.6 | ND | ND | ND | ND |
| IPM | 31 | 3.2 | 0.0 | 96.8 | ND | ND | ND | ND |
| MEM | 35 | 2.9 | 0.0 | 97.1 | ND | ND | ND | ND |
| AMK | 32 | 3.1 | 0.0 | 96.9 | ND | ND | ND | ND |
| GEN | 31 | 3.2 | 3.2 | 93.5 | ND | ND | ND | ND |
| CIP | 35 | 14.3 | 5.7 | 80.0 | ND | ND | ND | ND |
| SXT | 15 | 13.3 | 0.0 | 86.7 | ND | ND | ND | ND |
| *A. baumannii* | | | | | | | | |
| SAM | 36 | 66.7 | 25.0 | 8.3 | 28 | 42.9 | 14.3 | 42.9 |
| TZP | 38 | 94.7 | 0.0 | 5.3 | ND | ND | ND | ND |
| FEP | 42 | 50.0 | 42.9 | 7.1 | 28 | 35.7 | 25.0 | 39.3 |
| IPM | 38 | 94.7 | 0.0 | 5.3 | 13 | 84.6 | 0.0 | 15.4 |
| MEM | 42 | 92.9 | 2.4 | 4.8 | 28 | 53.6 | 0.0 | 46.4 |
| AMK | 21 | 57.1 | 19.0 | 23.8 | 18 | 33.3 | 0.0 | 66.7 |
| GEN | 30 | 80.0 | 16.7 | 3.3 | 28 | 42.9 | 7.1 | 50.0 |
| CIP | 42 | 95.2 | 0.0 | 4.8 | 28 | 53.6 | 0.0 | 46.4 |
| *P. aeruginosa* | | | | | | | | |
| CZT | 61 | 16.4 | 3.3 | 80.3 | 18 | 11.1 | 5.6 | 83.3 |
| TZP | 101 | 29.7 | 7.9 | 62.4 | 43 | 34.9 | 7.0 | 58.1 |
| CAZ | 118 | 23.7 | 5.1 | 71.2 | 61 | 39.3 | 6.6 | 54.1 |
| FEP | 118 | 16.1 | 11.0 | 72.9 | 60 | 36.7 | 6.7 | 56.7 |
| IPM | 110 | 38.2 | 3.6 | 58.2 | 45 | 37.8 | 6.7 | 55.6 |
| MEM | 118 | 34.7 | 2.5 | 62.7 | 61 | 45.9 | 3.3 | 50.8 |
| CIP | 118 | 31.4 | 5.1 | 63.5 | ND | ND | ND | ND |

CZT: Ceftolozane/Tazobactam, AMP: Ampicillin, AMC: Amoxicillin/Clavulanic acid, CZA: Ceftazidime/Avibactam, SAM: Ampicillin/Sulbactam, CAZ: Ceftazidime, CRO: Ceftriaxone, FEP: Cefepime, FOX: Cefoxitin, ATM: Aztreonam, ETP: Ertapenem, IPM: Imipenem, MEM: Meropenem, AMK: Amikacin, GEN: Gentamicin, CIP: Ciprofloxacin, SXT: Sulfamethoxazole/Trimethoprim, TZP: Piperacillin/Tazobactam. ND: Not Determined.
