# Supplementary material for: The threat of multidrug-resistant microorganisms: active surveillance of key antimicrobial resistant pathogens in 2025 - a report from the INVIFAR network
Source: Eur J Clin Microbiol Infect Dis. 2026 Jan 6;45(4):1041–57. doi: 10.1007/s10096-025-05330-2 (PMC13086762; doi:10.1007/s10096-025-05330-2)
Supplement: Supplementary file 6 — Supplementary Material 6 [file 10096_2025_5330_MOESM6_ESM.docx]

Suppl Table 6. Distribution of antibiotic resistance for *S. aureus* according to site of attention, clinical specimen and age group.

| **Antibiotic** | **n** | **%R** | **%I** | **%S** | **n** | **%R** | **%I** | **%S** | **n** | **%R** | **%I** | **%S** | **n** | **%R** | **%I** | **%S** | **p** |
| --- | --- | --- | --- | --- | --- | --- | --- | --- | --- | --- | --- | --- | --- | --- | --- | --- | --- |
|  | **EME** | | | | **ICU** | | | | **INX** | | | | **OUT** | | | |  |
| OXA | 178 | 23.0 | 0.0 | 77.0 | 131 | 20.6 | 0.0 | 79.4 | 760 | 23.4 | 0.0 | 76.6 | 163 | 14.7 | 0.0 | 85.3 | 0.103 |
| FOX | ND | ND | ND | ND | ND | ND | ND | ND | 32 | 15.6 | 0.0 | 84.4 | 20 | 25.0 | 0.0 | 75.0 | ND |
| GEN | 28 | 7.1 | 0.0 | 92.9 | 26 | 30.8 | 0.0 | 69.2 | 122 | 13.9 | 0.8 | 85.2 | 53 | 28.3 | 0.0 | 71.7 | **0.019** |
| CIP | 169 | 20.7 | 3.6 | 75.7 | 125 | 15.2 | 4.0 | 80.8 | 728 | 23.5 | 1.8 | 74.7 | 181 | 17.1 | 3.3 | 79.6 | 0.107 |
| LVX | 171 | 21.6 | 1.2 | 77.2 | 118 | 19.5 | 0.8 | 79.7 | 703 | 23.3 | 0.3 | 76.4 | 163 | 17.2 | 0.6 | 82.2 | 0.351 |
| SXT | 179 | 5.6 | 0.0 | 94.4 | 136 | 2.2 | 0.0 | 97.8 | 776 | 5.7 | 0.0 | 94.3 | 180 | 8.9 | 0.0 | 91.1 | 0.092 |
| CLI | 176 | 25.0 | 1.1 | 73.9 | 135 | 23.0 | 0.7 | 76.3 | 764 | 27.9 | 0.3 | 71.9 | 161 | 26.7 | 0.0 | 73.3 | 0.653 |
| DAP | ND | ND | ND | ND | 108 | 0.9 | 0.0 | 99.1 | ND | ND | ND | ND | ND | ND | ND | ND | ND |
| ERY | 177 | 25.4 | 2.8 | 71.8 | 135 | 21.5 | 1.5 | 77.0 | 768 | 26.8 | 3.9 | 69.3 | 161 | 18.6 | 3.7 | 77.6 | 0.103 |
| AZM | 19 | 47.4 | 5.3 | 47.4 | ND | ND | ND | ND | 82 | 39.0 | 0.0 | 61.0 | 30 | 23.3 | 3.3 | 73.3 | 0.174 |
| LNZ | 177 | 0.0 | 0.0 | 100.0 | 136 | 0.7 | 0.0 | 99.3 | 766 | 0.5 | 0.0 | 99.5 | 162 | 1.9 | 0.0 | 98.1 | 0.170 |
| TCY | 157 | 3.8 | 0.0 | 96.2 | 106 | 1.9 | 0.0 | 98.1 | 658 | 3.5 | 0.3 | 96.2 | 150 | 6.7 | 0.7 | 92.7 | 0.202 |
| QDA | ND | ND | ND | ND | 18 | 0.0 | 0.0 | 100.0 | 70 | 7.1 | 0.0 | 92.9 | 28 | 7.1 | 0.0 | 92.9 | ND |
|  | **Urine** | | | | **Respiratory** | | | | **Blood** | | | | **Abscess** | | | |  |
| OXA | 77 | 6.5 | 0.0 | 93.5 | 287 | 21.3 | 0.0 | 78.7 | 276 | 25.7 | 0.0 | 74.3 | 112 | 23.2 | 0.0 | 76.8 | **0.004** |
| FOX | ND | ND | ND | ND | 14 | 0.0 | 0.0 | 100.0 | 24 | 16.7 | 0.0 | 83.3 | ND | ND | ND | ND | ND |
| GEN | 19 | 15.8 | 0.0 | 84.2 | 48 | 14.6 | 0.0 | 85.4 | 70 | 18.6 | 0.0 | 81.4 | 19 | 21.1 | 0.0 | 78.9 | 0.909 |
| CIP | 78 | 9.0 | 0.0 | 91.0 | 239 | 18.0 | 1.3 | 80.8 | 286 | 25.5 | 4.2 | 70.3 | 114 | 21.1 | 2.6 | 76.3 | **0.004** |
| LVX | 73 | 6.8 | 0.0 | 93.2 | 273 | 20.9 | 0.0 | 79.1 | 269 | 24.5 | 1.1 | 74.3 | 101 | 20.8 | 0.0 | 79.2 | **0.011** |
| SXT | 79 | 5.1 | 0.0 | 94.9 | 297 | 4.4 | 0.0 | 95.6 | 287 | 8.0 | 0.0 | 92.0 | 115 | 8.7 | 0.0 | 91.3 | 0.212 |
| CLI | 71 | 12.7 | 0.0 | 87.3 | 291 | 27.5 | 0.3 | 72.2 | 279 | 25.4 | 0.7 | 73.8 | 115 | 20.0 | 1.7 | 78.3 | **0.044** |
| DAP | ND | ND | ND | ND | 206 | 0.5 | 1.0 | 98.5 | ND | ND | ND | ND | ND | ND | ND | ND | ND |
| ERY | 72 | 2.8 | 4.2 | 93.1 | 290 | 26.6 | 1.7 | 71.7 | 281 | 23.5 | 4.3 | 72.2 | 115 | 22.6 | 6.1 | 71.3 | **<0.001** |
| AZM | ND | ND | ND | ND | 23 | 60.9 | 0.0 | 39.1 | 45 | 31.1 | 0.0 | 68.9 | 13 | 38.5 | 15.4 | 46.2 | 0.060 |
| LNZ | 78 | 0.0 | 0.0 | 100.0 | 288 | 1.0 | 0.0 | 99.0 | 276 | 0.0 | 0.0 | 100.0 | 113 | 0.0 | 0.0 | 100.0 | 0.131 |
| TCY | 70 | 2.9 | 0.0 | 97.1 | 203 | 4.4 | 0.5 | 95.1 | 237 | 3.8 | 0.0 | 96.2 | 105 | 10.5 | 0.0 | 89.5 | 0.031 |
|  | **0-18 y** | | | | **19-59 y** | | | | **≥ 60 y** | | | |  | | | |  |
| OXA | 142 | 15.5 | 0.0 | 84.5 | 740 | 23.0 | 0.0 | 77.0 | 313 | 22.4 | 0.0 | 77.6 | ND | ND | ND | ND | 0.139 |
| FOX | 11 | 9.1 | 0.0 | 90.9 | 74 | 33.8 | 2.7 | 63.5 | 50 | 36.0 | 2.0 | 62.0 | ND | ND | ND | ND | 0.199 |
| GEN | 21 | 14.3 | 0.0 | 85.7 | 119 | 17.6 | 0.8 | 81.5 | 83 | 21.7 | 0.0 | 78.3 | ND | ND | ND | ND | 0.669 |
| CIP | 140 | 8.6 | 4.3 | 87.1 | 719 | 23.5 | 2.2 | 74.3 | 309 | 23.0 | 2.6 | 74.4 | ND | ND | ND | ND | **<0.001** |
| LVX | 126 | 7.1 | 0.8 | 92.1 | 695 | 23.6 | 0.3 | 76.1 | 303 | 24.4 | 1.0 | 74.6 | ND | ND | ND | ND | **<0.001** |
| SXT | 145 | 11.7 | 0.0 | 88.3 | 759 | 4.3 | 0.0 | 95.7 | 330 | 6.7 | 0.0 | 93.3 | ND | ND | ND | ND | **0.002** |
| CLI | 142 | 16.9 | 0.0 | 83.1 | 738 | 28.0 | 0.4 | 71.5 | 321 | 28.3 | 0.6 | 71.0 | ND | ND | ND | ND | **0.016** |
| DAP | 106 | 0.0 | 1.9 | 98.1 | ND | ND | ND | ND | ND | ND | ND | ND | ND | ND | ND | ND | ND |
| ERY | 143 | 16.1 | 4.2 | 79.7 | 742 | 26.0 | 3.4 | 70.6 | 322 | 28.0 | 3.4 | 68.6 | ND | ND | ND | ND | **0.022** |
| AZM | ND | ND | ND | ND | 74 | 35.1 | 2.7 | 62.2 | 52 | 48.1 | 0.0 | 51.9 | ND | ND | ND | ND | 0.181 |
| LNZ | 140 | 0.0 | 0.0 | 100.0 | 743 | 0.5 | 0.0 | 99.5 | 322 | 1.2 | 0.0 | 98.8 | ND | ND | ND | ND | 0.253 |
| TCY | 102 | 6.9 | 1.0 | 92.2 | 670 | 2.4 | 0.3 | 97.3 | 269 | 6.3 | 0.0 | 93.7 | ND | ND | ND | ND | **0.004** |
| TEC | ND | ND | ND | ND | ND | ND | ND | ND | 13 | 0.0 | 0.0 | 100.0 | ND | ND | ND | ND | 0.421 |
| QDA | ND | ND | ND | ND | 72 | 4.2 | 0.0 | 95.8 | 49 | 8.2 | 0.0 | 91.8 | ND | ND | ND | ND | 0.355 |

OXA: Oxacillin, FOX: Cefoxitin, GEN: Gentamicin, CIP: Ciprofloxacin, LVX: Levofloxacin, SXT: Sulfamethoxazole/Trimethoprim, CLI: Clindamycin, DAP: Daptomycin, ERY: Erythromycin, AZM: Azithromycin, LNZ: Linezolid, TCY: Tetracycline, QDA: Quinupristin/Dalfopristin, TEC: Teicoplanin, Not Determined, y: years.
