# Supplementary material for: The threat of multidrug-resistant microorganisms: active surveillance of key antimicrobial resistant pathogens in 2025 - a report from the INVIFAR network
Source: Eur J Clin Microbiol Infect Dis. 2026 Jan 6;45(4):1041–57. doi: 10.1007/s10096-025-05330-2 (PMC13086762; doi:10.1007/s10096-025-05330-2)
Supplement: Supplementary file 7 — Supplementary Material 7 [file 10096_2025_5330_MOESM7_ESM.docx]

Suppl Table 7. Antibiotic resistance for *E. faecium* according to clinical specimen

|  | **Urine** | | | | **Blood** | | | | **Abscess** | | | | **Biopsies** | | | | **p** |
| --- | --- | --- | --- | --- | --- | --- | --- | --- | --- | --- | --- | --- | --- | --- | --- | --- | --- |
| **Antibiotic** | **n** | **%R** | **%I** | **%S** | **n** | **%R** | **%I** | **%S** | **n** | **%R** | **%I** | **%S** | **n** | **%R** | **%I** | **%S** |  |
| PEN | 113 | 89.4 | 0.0 | 10.6 | 47 | 70.2 | 0 | 29.8 | 27 | 81.5 | 0.0 | 18.5 | 45 | 68.9 | 0.0 | 31.1 | ND |
| AMP | 113 | 89.4 | 0.0 | 10.6 | 46 | 67.4 | 0 | 32.6 | 27 | 81.5 | 0.0 | 18.5 | 49 | 71.4 | 0.0 | 28.6 | **0.004** |
| CIP | 107 | 73.8 | 9.3 | 16.8 | 41 | 51.2 | 9.8 | 39 | 25 | 80.0 | 4.0 | 16.0 | 43 | 46.5 | 18.6 | 34.9 | **0.003** |
| LVX | 102 | 70.6 | 2.9 | 26.5 | 39 | 46.2 | 7.7 | 46.2 | 23 | 73.9 | 4.3 | 21.7 | 39 | 48.7 | 2.6 | 48.7 | **0.010** |
| ERY | 112 | 86.6 | 9.8 | 3.6 | 45 | 77.8 | 8.9 | 13.3 | 27 | 88.9 | 7.4 | 3.7 | 45 | 77.8 | 17.8 | 4.4 | 0.113 |
| LNZ | 113 | 3.5 | 1.8 | 94.7 | 46 | 2.2 | 4.3 | 93.5 | 27 | 3.7 | 11.1 | 85.2 | 49 | 2.0 | 0.0 | 98.0 | 0.925 |
| VAN | 113 | 51.3 | 0.0 | 48.7 | 47 | 31.9 | 0 | 68.1 | 27 | 55.6 | 0.0 | 44.4 | 48 | 22.9 | 2.1 | 75.0 | **0.002** |
| TCY | 101 | 30.7 | 2.0 | 67.3 | 30 | 36.7 | 0 | 63.3 | 24 | 37.5 | 8.3 | 54.2 | 24 | 41.7 | 0.0 | 58.3 | ND |
| GEH | 98 | 32.7 | 0.0 | 67.3 | ND | ND | ND | ND | ND | ND | ND | ND | ND | ND | ND | ND | ND |
| STH | 92 | 12.0 | 0.0 | 88.0 | ND | ND | ND | ND | ND | ND | ND | ND | ND | ND | ND | ND | ND |
| NIT | 104 | 51.0 | 31.7 | 17.3 | ND | ND | ND | ND | ND | ND | ND | ND | ND | ND | ND | ND | ND |

PEN: Penicillin, AMP: Ampicillin, CIP: Ciprofloxacin, LVX: Levofloxacin, ERY: Erythromycin, LNZ: Linezolid, VAN: Vancomycin, TCY: Tetracycline, GEH: Gentamicin High-dose, STH: Streptomycin High-dose, NIT: Nitrofurantoin.ND: Not Determined.
