# Supplementary material for: The threat of multidrug-resistant microorganisms: active surveillance of key antimicrobial resistant pathogens in 2025 - a report from the INVIFAR network
Source: Eur J Clin Microbiol Infect Dis. 2026 Jan 6;45(4):1041–57. doi: 10.1007/s10096-025-05330-2 (PMC13086762; doi:10.1007/s10096-025-05330-2)
Supplement: Supplementary file 8 — Supplementary Material 8 [file 10096_2025_5330_MOESM8_ESM.docx]

Suppl Table 8. Distribution of antibiotic resistance by gender

|  | **Female** | | | | **Male** | | | |  |
| --- | --- | --- | --- | --- | --- | --- | --- | --- | --- |
| **Antibiotic** | **n** | **%R** | **%I** | **%S** | **n** | **%R** | **%I** | **%S** | **p** |
| *E. coli* | | | | | | | | | |
| SAM | 3,875 | 35.5 | 17.7 | 46.8 | 2,005 | 48.7 | 15.3 | 36.0 | **<0.001** |
| CAZ | 4,134 | 27.6 | 11.1 | 61.3 | 2,106 | 43.4 | 15.3 | 41.4 | **<0.001** |
| CRO | 4,176 | 46.0 | 0.2 | 53.7 | 2,047 | 65.6 | 0.2 | 34.2 | **<0.001** |
| FEP | 4,131 | 31.5 | 7.6 | 60.9 | 2,114 | 47.9 | 11.2 | 40.9 | **<0.001** |
| ETP | 4,143 | 1.9 | 0.7 | 97.4 | 2,134 | 4.6 | 0.7 | 94.7 | **<0.001** |
| IPM | 1,344 | 2.5 | 0.7 | 96.7 | 830 | 4.6 | 0.6 | 94.8 | **0.010** |
| MEM | 4,180 | 1.4 | 0.1 | 98.5 | 2,142 | 3.6 | 0.4 | 96.0 | **<0.001** |
| SXT | 3,683 | 53.5 | 0.0 | 46.5 | 1,665 | 56.6 | 0.0 | 43.4 | **0.034** |
| *K. pneumoniae* | | | | | | | | | |
| CZA | 106 | 4.7 | 0.0 | 95.3 | 109 | 6.4 | 0.0 | 93.6 | 0.586 |
| SAM | 647 | 38.3 | 7.1 | 54.6 | 687 | 51.4 | 7.3 | 41.3 | **<0.001** |
| CAZ | 677 | 29.1 | 11.5 | 59.4 | 716 | 40.2 | 15.1 | 44.7 | **<0.001** |
| CRO | 690 | 44.5 | 0.1 | 55.4 | 691 | 57.7 | 0.0 | 42.3 | **<0.001** |
| FEP | 687 | 32.3 | 5.1 | 62.6 | 729 | 45.4 | 4.3 | 50.3 | **<0.001** |
| FOX | 119 | 11.8 | 3.4 | 84.9 | 104 | 17.3 | 2.9 | 79.8 | 0.244 |
| ATM | 108 | 45.4 | 0.9 | 53.7 | 128 | 55.5 | 1.6 | 43.0 | 0.108 |
| ETP | 693 | 4.3 | 1.0 | 94.7 | 733 | 7.4 | 1.1 | 91.5 | **0.015** |
| IPM | 300 | 5.3 | 1.0 | 93.7 | 340 | 6.5 | 1.5 | 92.1 | 0.533 |
| MEM | 705 | 3.3 | 0.0 | 96.7 | 735 | 6.4 | 0.5 | 93.1 | **0.005** |
| SXT | 582 | 44.3 | 0.2 | 55.5 | 543 | 57.3 | 0.0 | 42.7 | **<0.001** |
| *A. baumannii* | | | | | | | | | |
| SAM | 150 | 50.7 | 20.7 | 28.7 | 241 | 48.5 | 17.8 | 33.6 | 0.399 |
| FEP | 155 | 45.8 | 25.8 | 28.4 | 242 | 44.6 | 25.6 | 29.8 | 0.766 |
| IPM | 110 | 75.5 | 0.0 | 24.5 | 178 | 68.5 | 0.0 | 31.5 | 0.208 |
| MEM | 157 | 70.7 | 0.0 | 29.3 | 244 | 66.4 | 1.6 | 32.0 | 0.501 |
| GEN | 145 | 51.7 | 14.5 | 33.8 | 224 | 50.9 | 13.4 | 35.7 | 0.760 |
| CIP | 158 | 70.3 | 2.5 | 27.2 | 244 | 70.9 | 0.4 | 28.7 | 0.849 |
| *P. aeruginosa* | | | | | | | | | |
| CZT | 149 | 16.1 | 3.4 | 80.5 | 200 | 8.0 | 2.0 | 90.0 | **0.016** |
| CZA | 205 | 21.0 | 0.0 | 79.0 | 247 | 20.2 | 0.0 | 79.8 | 0.848 |
| TZP | 408 | 31.9 | 4.9 | 63.2 | 501 | 25.3 | 7.4 | 67.3 | 0.052 |
| CAZ | 568 | 27.5 | 4.0 | 68.5 | 743 | 25.6 | 4.0 | 70.4 | 0.437 |
| FEP | 561 | 19.3 | 9.4 | 71.3 | 744 | 16.9 | 10.8 | 72.3 | 0.332 |
| IPM | 431 | 35.7 | 5.8 | 58.5 | 522 | 31.6 | 3.6 | 64.8 | 0.107 |
| MEM | 567 | 29.3 | 6.2 | 64.6 | 742 | 29.5 | 5.5 | 65.0 | 0.989 |
| AMK | 271 | 13.7 | 3.3 | 83.0 | 366 | 20.5 | 3.8 | 75.7 | **0.023** |
| *S. aureus* | | | | | | | | | |
| OXA | 499 | 21.0 | 0.0 | 79.0 | ND | ND | ND | ND | ND |
| FOX | ND | ND | ND | ND | 39 | 15.4 | 0.0 | 84.6 | ND |
| CIP | 470 | 20.4 | 2.1 | 77.4 | 727 | 21.9 | 2.8 | 75.4 | 0.513 |
| LVX | 468 | 20.7 | 0.2 | 79.1 | 681 | 22.5 | 0.7 | 76.8 | 0.454 |
| SXT | 510 | 7.1 | 0.0 | 92.9 | 753 | 4.8 | 0.0 | 95.2 | 0.087 |
| CLI | 497 | 27.0 | 0.6 | 72.4 | 734 | 26.4 | 0.3 | 73.3 | 0.809 |
| ERY | 498 | 24.5 | 5.0 | 70.5 | 738 | 25.9 | 2.4 | 71.7 | 0.778 |
| LNZ | 503 | 0.8 | 0.0 | 99.2 | 732 | 0.5 | 0.0 | 99.5 | 0.592 |
| TCY | 413 | 3.9 | 0.0 | 96.1 | 654 | 3.8 | 0.5 | 95.7 | 0.978 |

CZT: Ceftolozane/Tazobactam, CZA: Ceftazidime/Avibactam, SAM: Ampicillin/Sulbactam, CAZ: Ceftazidime, CRO: Ceftriaxone, FEP: Cefepime, FOX: Cefoxitin, ATM: Aztreonam, ETP: Ertapenem, IPM: Imipenem, MEM: Meropenem, AMK: Amikacin, GEN: Gentamicin, CIP: Ciprofloxacin, LVX: Levofloxacin, SXT: Sulfamethoxazole/Trimethoprim, TZP: Piperacillin/Tazobactam, OXA: Oxacillin, CLI: Clindamycin, ERY: Erythromycin, LNZ: Linezolid, TCY: Tetracycline, ND: Not Determined.
